# Design, Modeling, and Demonstration of a New Dual-Mode Back-Assist Exosuit with Extension Mechanism

**DOI:** 10.1017/wtc.2021.1

**Published:** 2021-03-24

**Authors:** Erik P. Lamers, Karl E. Zelik

**Affiliations:** 1 Department of Mechanical Engineering, Vanderbilt University, Nashville, Tennessee, United States.; 2 Department of Biomedical Engineering, Vanderbilt University, Nashville, Tennessee, United States.; 3 Department of Physical Medicine and Rehabilitation, Vanderbilt University, Nashville, Tennessee, United States.

**Keywords:** wearable assistive devices, occupational exoskeletons, lifting, biomechanical modeling, comfort

## Abstract

Occupational exoskeletons and exosuits have been shown to reduce muscle demands and fatigue for physical tasks relevant to a variety of industries (e.g. logistics, construction, manufacturing, military, healthcare). However, adoption of these devices into the workforce has been slowed by practical factors related to comfort, form-factor, weight, and not interfering with movement or posture. We previously introduced an un-motorized, low-profile, dual-mode exosuit comprised of textile and elastic materials to address these adoption barriers. Here we build upon this prior work by introducing an extension mechanism that increases the moment arm of the exosuit while in engaged mode, then collapses in disengaged mode to retain key benefits related to being lightweight, low-profile, and unobstructive. Here we demonstrate both analytically and empirically how this extensible exosuit concept can (i) reduce device-to-body forces (which can improve comfort for some users and situations), or (ii) increase the magnitude of torque assistance about the low back (which may be valuable for heavy-lifting jobs) without increasing shoulder or leg forces relative to the prior form-fitting exosuit. We also introduce a novel mode-switching mechanism, as well as a human-exosuit biomechanical model to elucidate how individual design parameters affect exosuit assistance torque and device-to-body forces. The proof-of-concept prototype, case study, and modeling work provide a foundation for understanding and implementing extensible exosuits for a broad range of applications. We envision promising opportunities to apply this new dual-mode extensible exosuit concept to assist heavy-lifting, to further enhance user comfort, and to address the unique needs of last-mile delivery workers.

## 1. Introduction

Occupational exoskeletons and exosuits have been developed for industrial applications such as manufacturing, construction, and material handling (Ferris et al., 2019), and have been demonstrated to reduce physical demands, muscle activity, and fatigue during a variety of tasks (de Looze et al., 2016). Despite the promising potential of these technologies to alleviate physical strain on workers, their adoption into industry has been slowed by practical factors such as comfort, weight, and form-factor (Baltrusch et al., 2018; Wolff et al., 2014). The challenge is that users are generally unwilling to adopt a wearable device if it is uncomfortable or if it protrudes out from their body in a way that is obstructive, unsafe or restricts movements needed to perform their job (Baltrusch et al., 2020; Yandell et al., 2019).

To overcome these adoption barriers exoskeleton developers have been exploring various ways to reduce physical interference, and discomfort, through improvements in mechanical design, device sizing, robotic control, material selection, and the physical human-device interface (Imamura et al., 2011; Toxiri et al., 2019; Yandell et al., 2019). The last five years in particular has seen rapid advances and abundant innovation in the design of occupational exoskeletons and exosuits (Nussbaum et al., 2019). For instance, we previously developed a back-assist exosuit that was lightweight and sufficiently low-profile to fit underneath clothing, and was primarily made of soft textile and elastic materials to minimize pressure points, discomfort, and movement interference. We also demonstrated its ability to reduce low-back muscle activity during lifting and bending tasks (Lamers et al., 2018), and to reduce the rate of muscle fatigue (Lamers et al., 2020). This exosuit (detailed in previous work (Lamers et al., 2018)) uses elastic bands along the back, which stretch when the user bends forward or crouches down, creating an assistive torque about the low-back and hips that offloads the lumbar and hip extensor muscles. In a variation of this exosuit design we integrated a mode-switching clutch (both manual and motorized versions), which allowed the user to quickly engage and disengage the exosuit assistance on demand (Lamers et al., 2017; Zelik et al., 2017). Users disengaged the exosuit to have full and unrestricted range of motion when assistance was not needed.

The prior exosuit was designed to fit close to the body and therefore had a relatively short moment arm (~8 cm) relative to the lumbosacral joint (hereafter referred to as the L5-S1 joint). To provide an assistive torque of 20 Newton · meters (Nm) with the exosuit would require approximately 250 Newtons (N) of device-to-body forces on the shoulders and legs. Although this is far below the force comfort limit observed on the shoulders and legs in a previous study (~600–1000 N, (Yandell et al., 2020)), we highlight two compelling cases here. First, there may be individuals who are particularly sensitive to shoulder or leg forces and for whom we may want to achieve the same 20 Nm assistive torque but with reduced device-to-body forces to ensure comfort. Second, there may be individuals who are comfortable with the nominal device-to-body forces, but who are engaged in heavy lifting, and would like to increase the magnitude of exosuit assistance (e.g. to 40 Nm), but maintain the same magnitude of device-to-body forces on the shoulders and legs.

One simple solution is to increase the moment arm of the exosuit by adding a spacer between the elastic band and the back or buttocks. In this configuration, assistive torque could be maintained while decreasing the force through the elastic bands and applied to the shoulders and legs. Alternatively, in this configuration, if force through the elastic bands is held constant (at 250 N) then the assistive torque about the low-back would be increased. Devices such as the Personal Lift Assist Device have implemented this style of design, and have demonstrated that this simple solution works as expected (Abdoli-E & Stevenson, 2008; Abdoli-Eramaki et al., 2007). However, this solution re-introduces the problem of form-factor: the device now protrudes out from the back or buttocks in a way that can interfere with movement, various postures (e.g. sitting), and the work environment.

**Figure 1.**
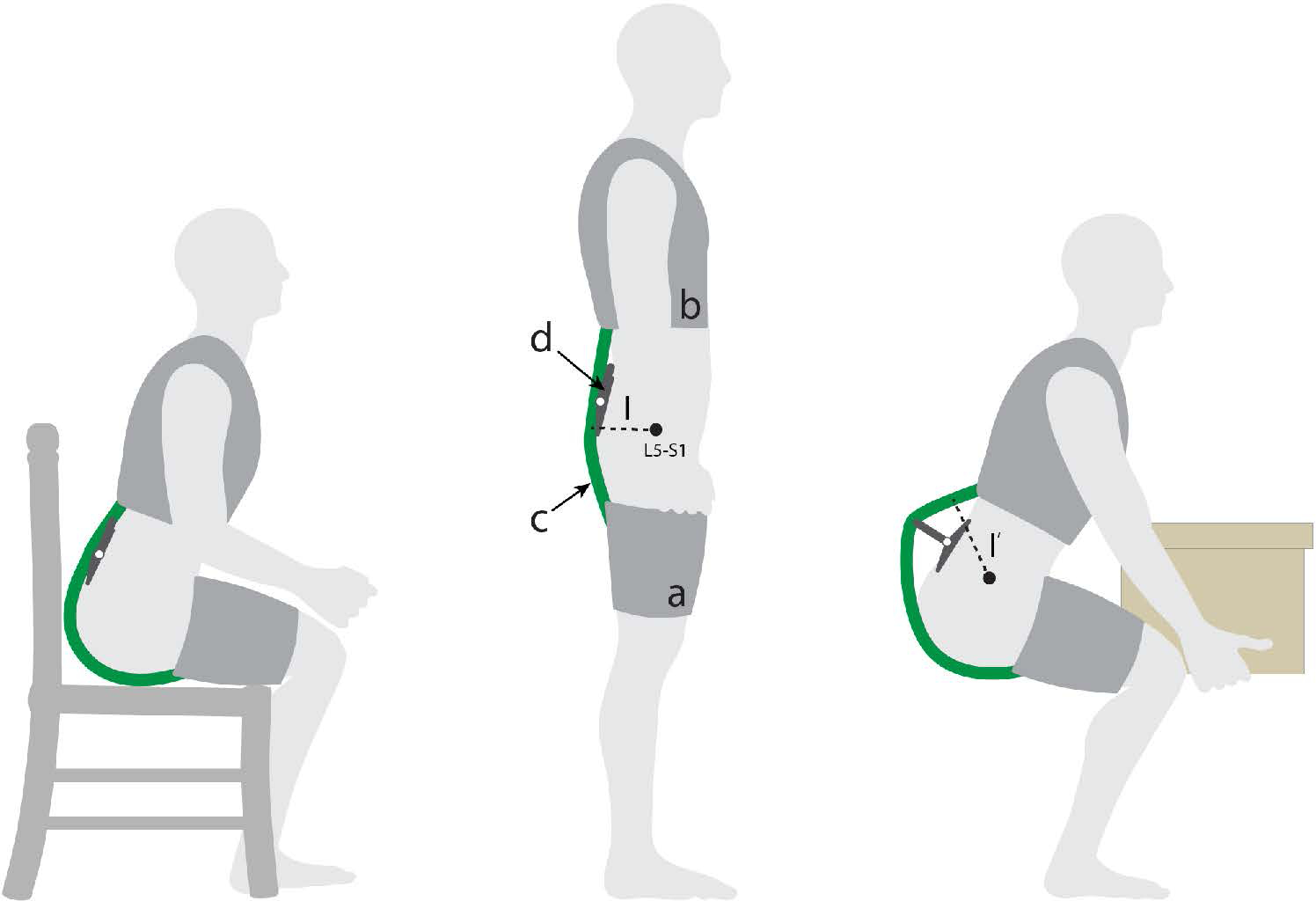
Conceptual depiction of the extensible exosuit. This concept is shown in disengaged (collapsed) mode during seated and standing postures, and in engaged (extended) mode during lifting. The extensible exosuit is composed of a leg (a) and trunk (b) interface, an elastic band (c), and a mechanism that can switch between an extended (larger moment arm *l*′) and collapsed state (smaller moment arm *l*). The elastic band (green) runs along the user’s posterior, over the moment arm mechanism, and connects the leg interface to the trunk interface. In engaged mode, as the user bends forward or crouches down, the elastic band stretches, applying tension forces to the leg and trunk interfaces. The addition of the extension mechanism redirects the path of the elastic band, increasing the exosuit moment arm (from l to l’) of the exosuit relative to the lumbosacral (L5-S1) joint. This simplified image is only intended to introduce the basic concept, and additional aspects of the design are detailed later in section 4).

**Figure 2.**
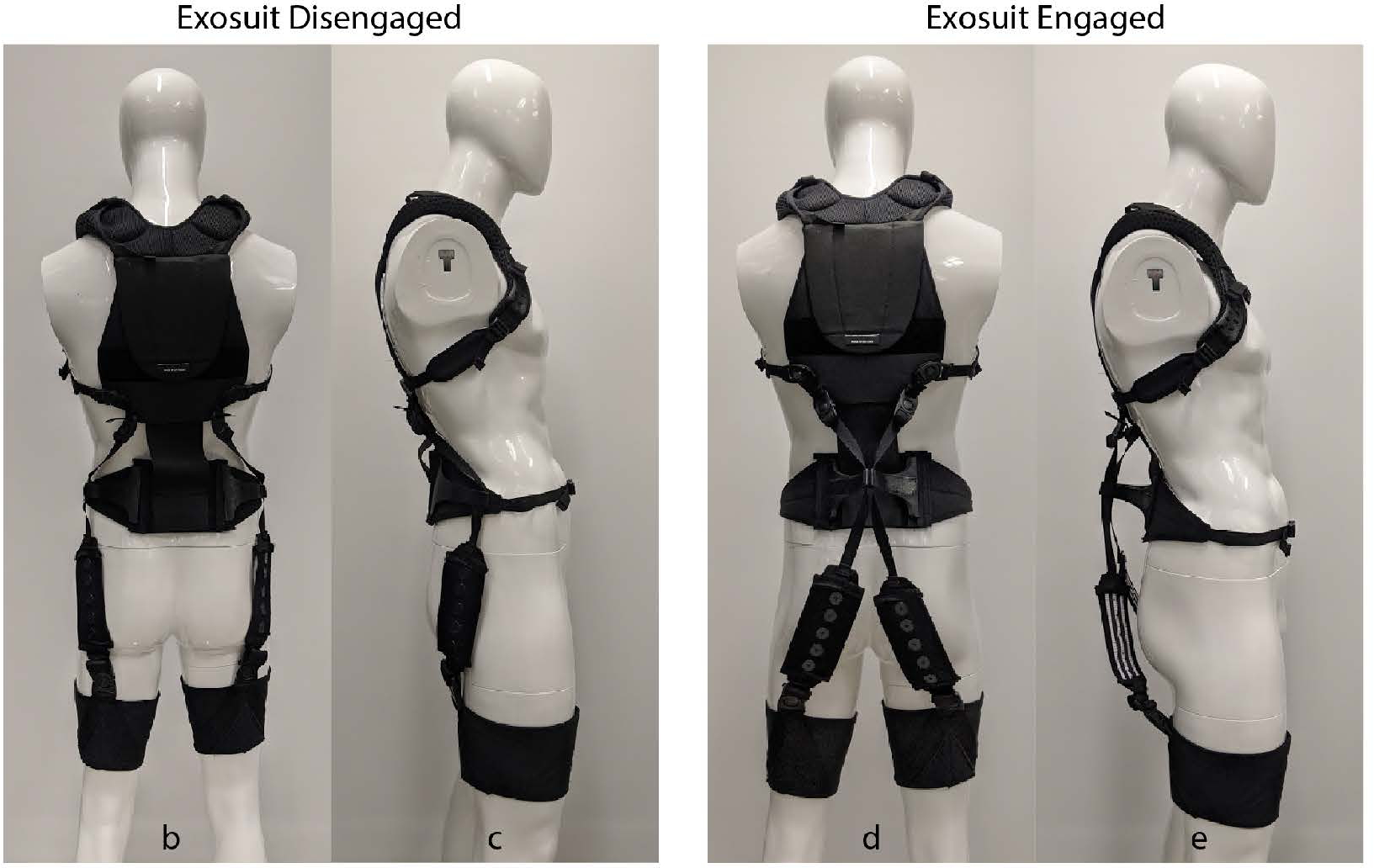
Photos of the extensible exosuit prototype in disengaged mode (two photos on the left), and in engaged mode (two photos on the right). Refer to the schematic in fig. 6 for call-outs to each component.

In this work we sought to model, develop and show proof-of-concept for a new patent-pending exosuit design (Zelik, Lamers, et al., 2020) that could temporarily increase the exosuit’s moment arm using an extension mechanism during lifting and bending tasks. The extension mechanism could then collapse and switch back to a low-profile configuration during unassisted tasks (e.g. walking, sitting, (fig. 1, left)) to avoid interfering with movement or the environment. The low-profile configuration is important because most of the time the primary goal of an exosuit is simply to not get in the way of the user. Even in jobs that are characterized by frequent or intensive lifting, workers spend only a fraction of their time bent over and lifting (e.g. ~ 10 percent of the time for retail workers, (Geissinger et al., 2020)) and are otherwise performing tasks which do not require exosuit assistance. For most situations and occupations we would not expect a temporary protrusion (e.g. an extension mechanism) from the back during lifting or bending to interfere with the task or surrounding environment. This is because generally when a person is executing a manual lifting or bending task, there is not another person or object immediately behind them or encroaching on their backside. We have found this to be true in our personal experiences and also observations of industrial workplaces such as warehouses, airports, distribution centers and construction sites. In this manuscript we detail computational modeling used to gain insight on exosuit design parameters, followed by design details on an exosuit prototype with an extension mechanism (fig. 2). We then present a case study demonstration of its function in engaged (assistive) mode with the mechanism extended, and in disengaged (stay-out-of-the-way) mode with the mechanism collapsed (fig. 1). For the remainder of this paper, we refer to the exosuit design detailed in our previous work as the *form-fitting exosuit*, and we refer to the newly proposed concept as the *extensible exosuit*.

## 2. Design Approach Overview

Our approach involved a sequence of biomechanical modeling (section 3), followed by prototype design (section 4), and then a proof-of-concept demonstration of an extensible exosuit prototype via a human subject case study (section 5). We developed a biomechanical exosuit-human model to gain insight on which design parameters were most important and how they interplay to affect device-to-body forces. Next we used these model insights to inform design parameter selection, and fabricated an exosuit prototype with an extension mechanism (fig. 2). Finally we performed a human subject case study to demonstrate mechanical function of the prototype. Specifically, we sought to confirm experimentally (i) that the extensible exosuit could provide the same L5-S1 joint torque assistance as the form-fitting exosuit but with lower device-to-body forces on the shoulders and legs, and (ii) that the extensible exosuit could remain sufficiently low-profile when it was disengaged such that it did not interfere with common movements and postures like walking and sitting.

## 3. Modeling

**Figure 3.**
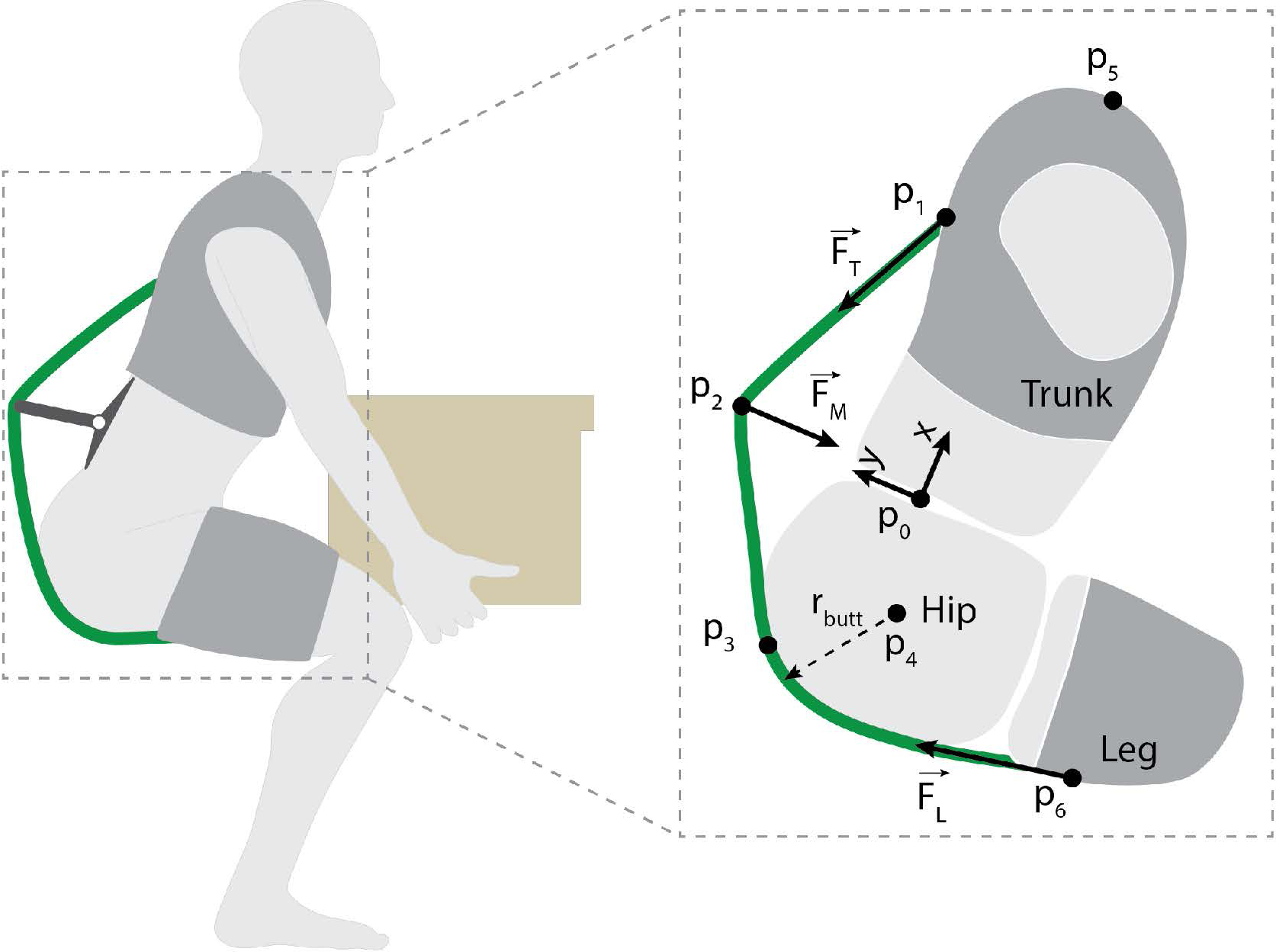
Static model of the exosuit-human system. The exosuit is comprised of a leg interface, a trunk interface, an elastic band (green curve) and an extension mechanism. The leg interface and trunk interface attach to the leg and trunk respectively, and are coupled by an elastic band. The exosuit creates an assistive torque by applying forces at the trunk $\left(\vec{F_{T}}\right)$ and waist $\left(\vec{F_{M}}\right)$ and legs $\left(\vec{F_{L}}\right)$. *p*_0_ is the location of the L5-S1 joint and coordinate system origin. *p*_1_ is the point at which the elastic band attaches to the trunk interface (and applies $\vec{F_{T}}$). *p*_2_ is the routing point for the elastic band on the extension mechanism (and where $\vec{F_{M}}$ is applied). Note that when *p*_2_ sits flush with the trunk/waist, there is no extension mechanism and the device behaves like the previous form-fitting exosuit detailed in (Lamers et al., 2018). *p*_3_ is the point at which the elastic band first makes contact with the posterior waist (simplified as a tangency point with a circle of radius *r*_*butt*_). *p*_4_ is the hip center of rotation, *p*_5_ is the top most point on the shoulder, and *p*_6_ is the anchoring point on the leg.

Previous biomechanical models of wearable back-assist devices (Abdoli-Eramaki et al., 2007; Imamura et al., 2011; Lamers et al., 2018; Toxiri et al., 2015) explain the underlying physics of how these devices offload the lumbar muscles and spine. We sought to build upon this prior work by characterizing how to adjust specific exosuit design parameters to affect device-to-body forces and the exosuit moment arm about the spine. The rationale for this modeling is readily apparent in fig. 3 where we note that there are number of inter-related design choices such as where to anchor to each body segment, where to place the base of the extension mechanism along the back, and how to select the extension length of the mechanism. The effects of and the interplay between these parameters on device-to-body forces was unknown, but important for us to understand in order to inform the design and fabrication of a prototype.

We therefore developed a model of the human and exosuit that estimates the device-to-body forces (fig. 3, $\vec{F_{T}}$, $\vec{F_{M}}$,$\vec{F_{L}}$) needed to create a desired torque about the L5-S1 joint (fig. 3, *p*_0_). The model is a static, sagittal plane model of the exosuit and human system. We use a static model for simplicity since the goal was general design insight, and since exosuit mass is low and inertial effects are negligible. The model only considers the sagittal plane because the majority of the biological lumbar moment and exosuit assistive torque (*τ*_*exo*_) are observed in the sagittal plane (Lamers et al., 2018), and these dynamics typically dominate even in the presence of twisting or other non-sagittal trunk motions (Gagnon & Gagnon, 1992). The model primarily considers the exosuit assistance torque created about the L5-S1 joint because it commonly experiences the highest flexion torques along the spine (Bogduk & Macintosh, 1984). The model considers the exosuit and human mechanics when the exosuit is engaged (i.e. extension mechanism is lengthened and elastic bands are under tension) and the user is leaning forward (as in fig. 3). We focus on the device-to-body forces at the trunk $\left(\vec{F_{T}}\right)$, legs $\left(\vec{F_{L}}\right)$ and waist (via the extension mechanism,$\vec{F_{M}}$) because we have noted from experience that these tend to be areas that are more sensitive to external loads. Whereas we were not concerned about the device-to-body force on the buttocks because this area can comfortably sustain external forces on the order of a body weight (e.g. during sitting), and the device-to-body forces from our exosuits are far below this magnitude. The human body is modeled as a series of linked rigid-body segments. In this modeling section, we also assume negligible friction and thus that the magnitude of tension is constant throughout the elastic element (i.e. the tension magnitude at the trunk, $\left\|\vec{F_{T}}\right\|$, is equal to the tension magnitude at the leg,$\left\|\vec{F_{L}}\right\|$). We supplement this model by adding a routing point (fig. 3, *p*_2_), which redirects the path of the elastic band (fig. 3, green curve) and introduces a device-to-body force $\left(\vec{F_{M}}\right)$. This routing point (which is modeled as a friction-less pulley) is the main element which alters the exosuit moment arm about the spine.

We identified design parameter candidates to manipulate, which included: routing point location along the spine, routing point offset from the skin surface, number of routing points, elastic band attachment point on the trunk interface and the elastic band attachment point on the leg interface. We narrowed the options (based on initial modeling findings, physical intuition and expected end-user applications and constraints) to three key parameters: the routing point position along the back (fig. 3, *x*_2_), the routing point offset normal to the back (fig. 3, *y*_2_), and the position of the elastic anchoring point on the trunk interface (fig. 3, *x*_1_). We note that the elastic band attachment point on the leg interface (fig. 3, *p*_6_) was not considered a key parameter for this particular exosuit design because it had negligible effects on the moment arm about the L5-S1 joint. This is evident in fig. 3 where it can be seen that in this body configuration, regardless of the location of this leg attachment point, the elastic band will run along the same path from the buttocks (*p*_3_) to the extension mechanism (*p*_2_) to the trunk (*p*_1_). Nevertheless, this leg interface attachment point is reintroduced in alternative exosuit designs, where it can be an important parameter (see section 6.3 and appendix A.4).

### 3.1. Model Development

The torque created about the L5-S1 joint (*τ*_*exo*_, in the z-axis coming out of the page) by the exosuit is:

(1) $$\tau_{exo}=\tau_{T}+\tau_{M}$$

Where *τ*_*T*_ is the torque created by the device-to-body trunk force vector $\left(\vec{F_{T}}\right)$, and *τ*_*M*_ is the torque contribution from the device-to-body force vector from the extension mechanism $\left(\vec{F_{M}}\right)$:

(2) $$\tau_{T}=\vec{r_{10}}\times \vec{F_{T}}=\left(\vec{r_{10}}\times \vec{u_{21}}\right)\cdot \left\|\vec{F_{T}}\right\|$$

(3) $$\tau_{M}=\vec{r_{20}}\times \vec{F_{M}}=\left(\vec{r_{20}}\times \left(\vec{u_{32}}+\vec{u_{12}}\right)\right)\cdot \left\|\vec{F_{T}}\right\|$$

In equation eq. (2), $\vec{r_{10}}$ is the position vector from *p*_0_ to *p*_1_, and $\vec{u_{21}}$ is the unit vector from *p*_1_ to *p*_2_, and $\left\|\vec{F_{T}}\right\|$ is the tension magnitude in the elastic band. In eq. (3), $\vec{r_{20}}$ is the position vector from *p*_0_ to *p*_2_, and $\vec{u_{32}}$ is the unit vector from *p*_2_ to *p*_3_, and $\vec{u_{12}}$ is the unit vector from *p*_2_ to *p*_1_. The device-to-body forces ($\vec{F_{T}}$ and $\vec{F_{M}}$) only create torque about the L5-S1 joint if their line-of-action intersects the trunk body segment (e.g. at a point >$p_{{\text{0}}_{x}}$). The trunk interface anchoring point *p*_1_ (and therefore $\vec{F_{T}}$) in this model is constrained to sit on the trunk above the L5-S1 joint and create an extension torque about *p*_0_ because $\vec{F_{T}}$ is applied by an elastic band that can generate tension but not compression force. An extension mechanism supports the routing point (represented by *p*_2_), and this mechanism is allowed to sit anywhere along the posterior side of the waist or trunk. This model assumes that the extension mechanism will only bear compression loads (i.e. no bending moments). Physically, this means that the extension mechanism is assumed to be co-linear with $\vec{F_{M}}$ and will be anchored at the location on the back where _$\vec{F_{M}}$_ intersects the back. Note that $\vec{F_{M}}$ only creates a flexion (clockwise) torque about *p*_0_ when $\vec{F_{M}}$ intersects the trunk above *p*_0_, but otherwise $\vec{F_{M}}$ does not create torque about *p*_0_.

After minor algebraic manipulations of eqs. (1) to (3) we can calculate the exosuit moment arm (*r*_*T*_) about the L5-S1 joint with eq. (4):

(4) $$r_{T}=\frac{\tau_{exo}}{\left\|\vec{F_{T}}\right\|}={\left(\vec{r_{10}}\times \vec{u_{21}}+\vec{r_{20}}\times \left(\vec{u_{32}}+\vec{u_{12}}\right)\right)}^{-1}$$

Where this moment arm moment arm (*r*_*T*_) represents the Euclidean (straight-line) distance between the L5-S1 joint (*p*_0_) and the line of action of the elastic band from *p*_1_ to *p*_2_. Also, take note that (*r*_*T*_) is inversely proportional to $\left\|\vec{F_{T}}\right\|$. This means that for a fixed magnitude of *τ*_*exo*_ increasing (or maximizing) the moment arm *r*_*T*_ is analytically equivalent to decreasing (or minimizing) the device-to-body forces on the legs and trunk $\left(\vec{F_{T}}\right)$.

eq. (5) below is an expression for the magnitude of the device-to-body force from the extension mechanism $\left\|\vec{F_{M}}\right\|$:

(5) $$\left\|\vec{F_{M}}\right\|=\left\|\vec{u_{32}}+\vec{u_{12}}\right\|\cdot \left\|\vec{F_{T}}\right\|=k_{R}\cdot \left\|\vec{F_{T}}\right\|$$

Where we note that *k*_*R*_ is the ratio of force magnitude on the extension mechanism to the trunk force magnitude in the elastic band.

### 3.2. Model Parameter Exploration

A parameter exploration was performed by systematically varying the exosuit design parameters and characterizing the effects on the exosuit moment arm and device-to-body forces. Using eqs. (4) and (5) we performed a series of parameter sweeps: varying the trunk anchoring point (*x*_1_), the extension mechanism position along the back (*x*_2_) and the extension mechanism offset from the back (*y*_2_) across their respective domains as determined from anthropometric tables. Anthropometric data were used to scale the model to a 50^*th*^ percentile male stature (table 1, (Gordon et al., 2016; Jackson et al., 1998)).

Our primary goal was to understand parameter combinations that increase the exosuit moment arm (*r*_*T*_), which as noted above and shown analytically in eq. (4), corresponds to decreasing device-to-body forces on the trunk and legs. Another way to conceptualize the exosuit moment arm (*r*_*T*_) is that it is the ratio of exosuit torque (*τ*_*exo*_) per elastic band tension $\left(\left\|\vec{F_{T}}\right\|\right)$; therefore increasing the moment arm means that the exosuit can provide more torque for the same tension (or alternatively the same torque for less tension). Our secondary goal was to understand parameter combinations that minimize the extension mechanism force itself $\left(\left\|\vec{F_{M}}\right\|\right)$, since this is an additional device-to-body force applied to the back or waist. Reducing $\left\|\vec{F_{M}}\right\|$ is achieved by reducing *k*_*R*_, which is the ratio of the extension mechanism force magnitude per tension magnitude. To inform our prototype design we were most interested in exosuit parameter combinations that resulted in a relatively large *r*_*T*_ but a relatively small *k*_*R*_. There is a trade-off between these two variables, such that it is not possible to simultaneously maximize one and minimize the other. Therefore, we performed a parameter exploration to quantitatively map out these trade-offs, and inform the exosuit design.

### 3.3. Key Model Findings

**Figure 4.**
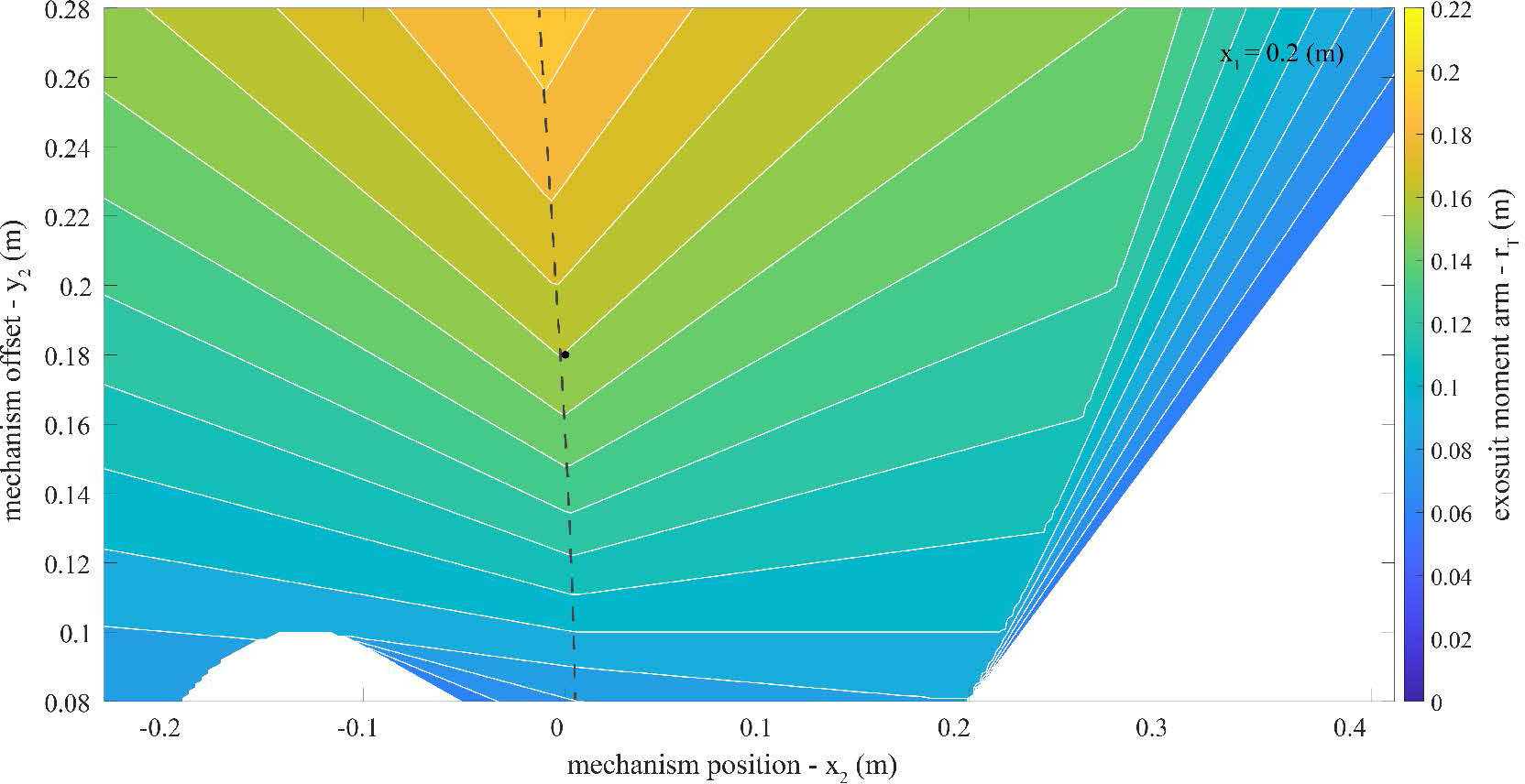
Extensible exosuit moment arm (*r*_*T*_) contour plot. Plotted is the extensible exosuit moment arm calculated with eq. (4). As a reminder, in this model higher values of *r*_*T*_ signify lower device-to-body forces on the shoulders and legs. This contour plot covers the parameter space of the extension mechanism location (*x*_2_) and offset (*y*_2_) specified in table 1, with a constant trunk interface anchoring point (*x*_1_ = 0.2 m). The target parameter combination selected for the proof-of-concept design in section 4.1 is plotted as a black dot (*x*_2_ = 0.0 m, *y*_2_ = 0.18 m). The dashed line represents extension mechanism parameter combinations (i.e. *x*_2_ and *y*_2_) with the smallest extension mechanism footprint (i.e. minimum *y*_2_) for a given *r*_*T*_ (i.e. contour line). Additional parameter exploration results which include the full range of *x*_1_ can be found in appendix A.2.

**Figure 5.**
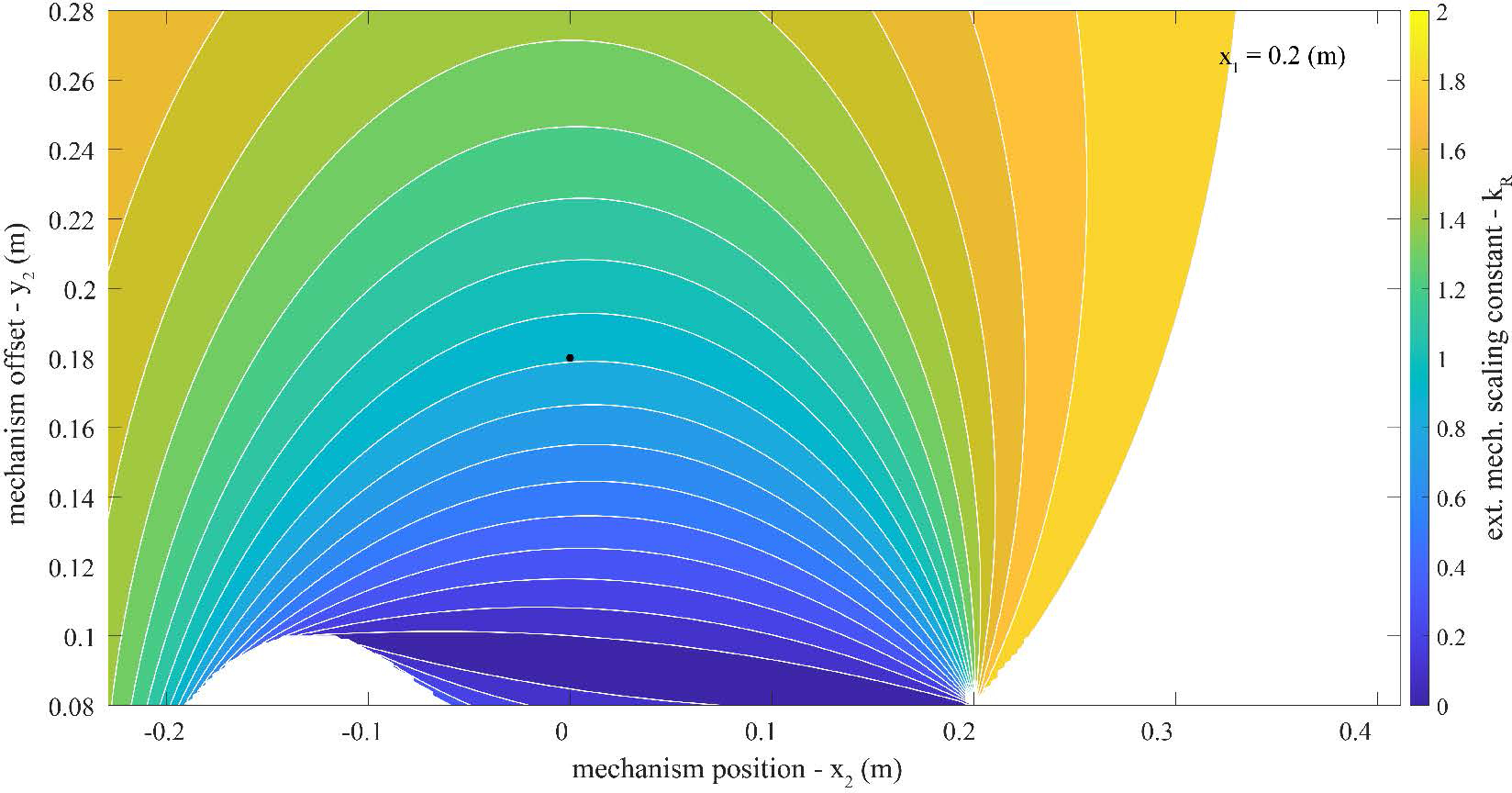
*k*_*R*_ contour plot. Plotted is the extension mechanism force scaling constant (*k*_*R*_) calculated with eq. (5). As a reminder, in this model lower values of *k*_*R*_ signify lower device-to-body forces from the extension mechanism onto the back or waist. This contour plot covers the parameter space of the extension mechanism location (*x*_2_) and offset (*y*_2_) specified in table 1, and a constant trunk interface anchoring point (*x*_1_ = 0.2 m). The parameter combination selected for the proof-of-concept design in section 4.1 is plotted as a black dot (*x*_2_ = 0.0 m, *y*_2_ = 0.18 m). Additional parameter exploration results which include the full range of *x*_1_ can be found in appendix A.2.

**Figure A.3.**
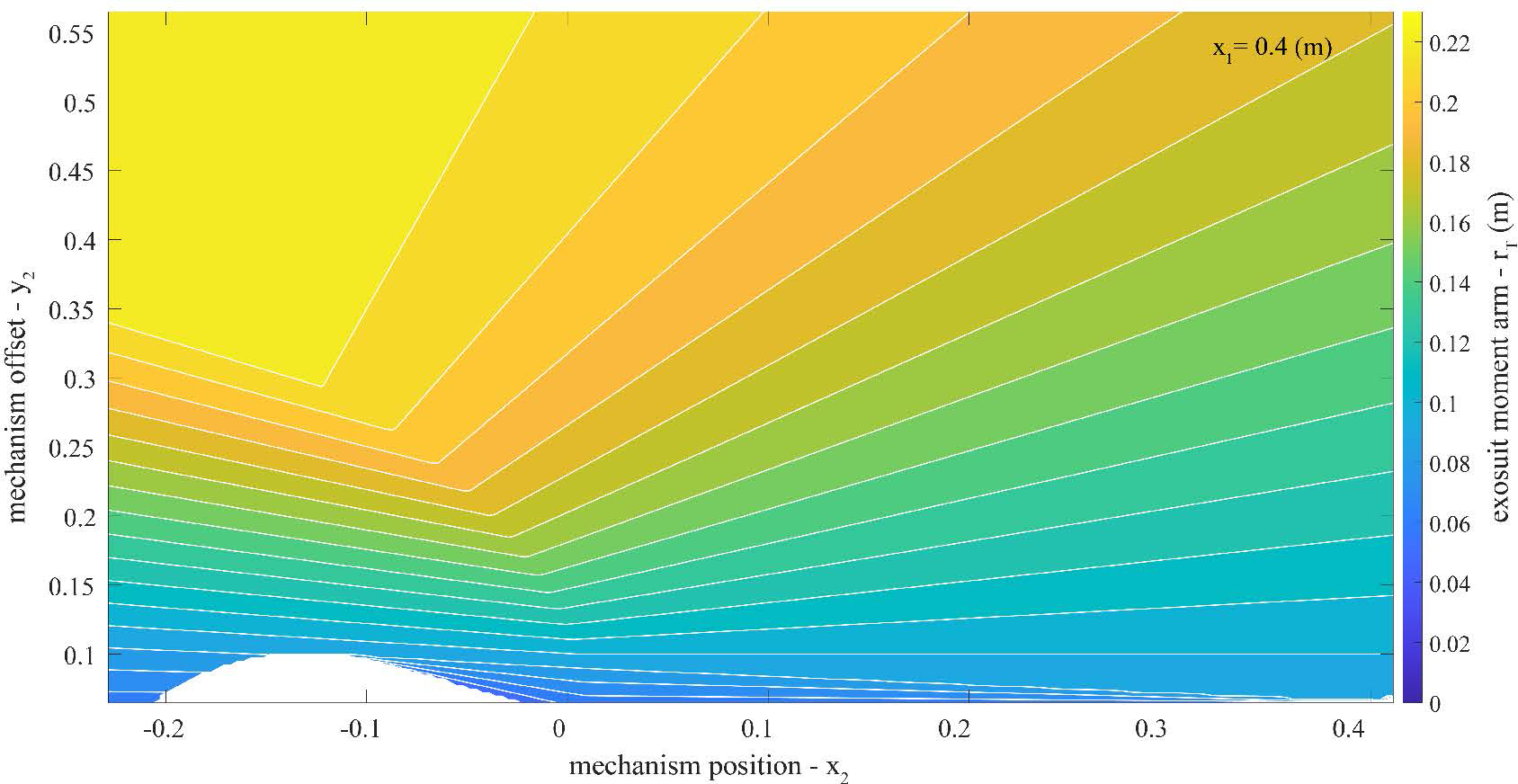
Extensible exosuit moment arm (*r*_*T*_) with *y*_2_ ranging from 0.1 m to 0.58 m calculated from eq. (4) across the *x*_2_ parameter domain specified in Table 1 and for a constant *x*_1_ = 0.4 m. We note here that although the mechanism offset (*y*_2_) continues to increase, the moment arm plateaus around 0.22 m (large yellow area in the top left of the plot). This suggests that for the specific proof-of-concept prototype explored here, the maximum moment arm is about 0.22 m. See section 6.4 for extended discussion of this topic.

The maximum exosuit moment arm *r*_*T*_ across the explored parameter space was 0.22 meters (m). For instance, this occurred when the extension mechanism was below the L5-S1 joint (*x*_2_ = −0.13 m), the extension mechanism was offset from the back (*y*_2_ = 0.28 m), and the elastic bands were attached to the trunk interface at the top of the shoulders (*x*_1_ = 0.41 m, fig. 4). The *k*_*R*_ at this parameter combination was 1.4 (fig. 5). We assumed a baseline *r*_*T*_ of of 0.08 m based on previous estimates (i.e. the approximate moment arm of the elastic band in our prior form-fitting exosuit (Lamers et al., 2018). Therefore, the maximum observed increase in *r*_*T*_ was 175% (0.08 to 0.22 m). Extended details about the exosuit parameters explored in this work can be found in appendix A.2. Below we briefly summarize the key findings used to inform prototype design:

- The main effect of the extension mechanism position (*x*_2_) was to change the location and orientation of the extension mechanism force vector along the back $\left(\vec{F_{M}}\right)$. The *x*_2_ value which resulted in the largest moment arm (*r*_*T*_) was near or slightly below the x-position of the L5-S1 joint (*x*_0_).
- The main effect of the extension mechanism offset (*y*_2_) was to change the moment arm (*r*_*T*_) and extension mechanism force magnitude (*k*_*R*_) where increasing *y*_2_ would increase both *r*_*T*_ and *k*_*R*_. However, increasing *y*_2_ beyond about 0.3 m had only minor effects on increasing the exosuit moment arm, which plateaued around 0.22 m (fig. A.3).
- The main effect of increasing the trunk interface anchoring point (*x*_1_) was to reduce the extension mechanism force magnitude $\left(\left\|\vec{F_{M}}\right\|\right)$; however, this effect (benefit) of increasing *x*_1_ plateaued around *x*_1_ = 0.2 m.

## 4. Design

### 4.1. Design Criteria

For the proof-of-concept prototype we aimed to design an extensible exosuit that would reduce $\left\|\vec{F_{T}}\right\|$ by about 50% and minimize the exosuit footprint (i.e. minimum extension mechanism offset *y*_2_) for the average male user (e.g. 50^*th*^ percentile). Using the model results, we followed the process detailed in appendix A.3 to choose appropriate exosuit design parameters (i.e. target design criteria) for the extensible exosuit proof-of-concept prototype as follows:

- The distance from the extension mechanism (and L5-S1) to the trunk interface anchoring point should be about 0.2 m (*x*_1_ = 0.2 m).
- The mechanism should sit approximately over the L5-S1 joint (*x*_2_ = 0.0 m).
- When engaged, the extension mechanism should be offset from the L5-S1 joint by about 0.18 m (*y*_2_ = 0.18 m).

### 4.2. Softgoods Design

**Figure 6.**
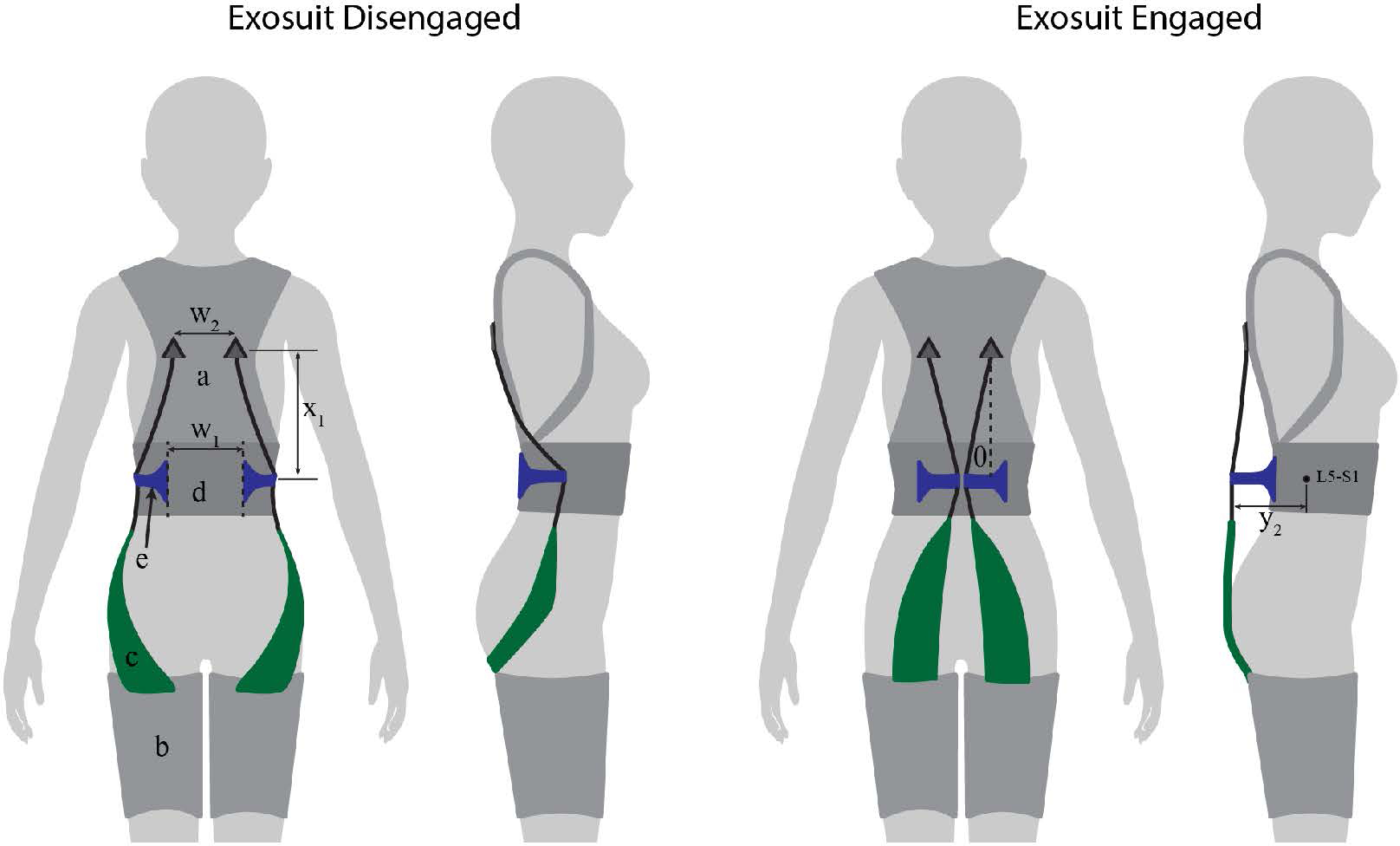
Extensible exosuit prototype schematic. This extensible exosuit design consists of a trunk interface (**a**), two leg interfaces (**b**), two elastic bands (**c**), a waist belt (**d**), and the extension mechanism flaps (**e**). The trunk interface is coupled with the leg interfaces via the elastic bands, which each consist of an elastic (green) and inelastic (black) segment in series. The elastic bands were routed through the flaps. **Exosuit disengaged:** the mechanism flaps (and the elastic bands) are folded to the user’s sides so that the elastic bands do not stretch or apply device-to-body forces during movement. **Exosuit engaged:** the mechanism flaps are folded to the users’ back (creating the offset *y*_2_) so that the elastic bands stretch and apply torque about the back and hips during tasks like lifting, bending, and stooping. The flaps rotate about hinges (dashed lines) which were spaced apart by 0.15 m (*w*_1_). The trunk interface anchoring points were spaced apart by 0.15 m as well (*w*_2_).

The extensible exosuit softgoods (i.e. textiles) consist of a trunk interface, two leg interfaces, and two elastic bands (fig. 6). The trunk interface includes breathable shoulder straps and a waist belt which are sewn together along the back. The shoulder straps (similar to backpack shoulder straps) transmit the trunk interface force to the users’ shoulders. The waist belt serves as a mounting point for the extension mechanism, and transmits a force at the users’ waist. The leg interfaces are conical fabric sleeves that transmit force to the user’s legs. The leg is shaped approximately like a conical frustum, which prevents the leg interfaces from migrating up the leg when upward forces are applied by the elastic bands. The elastic bands attach to the trunk interface about 0.2 m above the extension mechanism, according to the target parameters selected (fig. 6, *x*_1_). The elastic bands consist of fabric elastic (adapted from fabric resistance bands) sewn in series with non-stretch polyester webbing (fig. 6, c). The elastic bands are routed through the extension mechanism (fig. 6, e).

### 4.3. Extension Mechanism Design

The purpose of the extension mechanism is to move the elastic bands between two stable positions. In one position the mechanism and elastic bands should sit close to the body and the exosuit should be transparent to the user (i.e. not restrict or interfere with movement or posture). In the other position, the mechanism and elastic bands should be extended from the back (according to the exosuit parameters in section 4.1), and the elastic bands should stretch and apply torque about the L5-S1 joint as the user bends or lifts. Numerous extension mechanism designs exist, as this general class of mechanism has been used in robotics and prosthetics for creating variable stiffness actuation (e.g., Kim and Song, 2010; Kumar et al., 2020) and in a pneumatic balloon-actuated exoskeleton for generating assistive force (Inose et al., 2017)). For our current prototype development various design options were considered (e.g. four-bar mechanism, hinge (Zelik, Lamers, et al., 2020)). The benefits/drawbacks of each ultimately depend on the intended end-user and use case (making this more of a later-stage product development choice). The goal of this work was simply to demonstrate one embodiment of the concept, so we prioritized simplicity in form and function, and opted for a dual-flap, hinge-lever design, which we detail here.

The extension mechanism is made of two 3D printed flaps (fig. 6). Each flap attaches to the waist belt at about the L5-S1 level (target: *x*_2_ = 0.0 m). The flaps are 15 cm apart, centered over the mid-line of the spine (fig. 6, *w*_1_). Two elastic bands (one on the left and one on the right) are routed through each respective flap (fig. 6, e). The flaps are anchored to the waist belt with fabric hinges, which allow the flaps to rotate about an axis parallel to the spine. The flaps are designed to have a disengaged and an engaged mode. In disengaged mode, the extension mechanism flaps rest on the sides of the user’s waist (fig. 6, left). In engaged mode, the flaps are rotated to the posterior (bringing the elastic bands with them) until the flaps connect (held together via hook and loop), forming an offset from the L5-S1 (target: *y*_2_ = 0.18 m, (fig. 6, right). The elastic bands then stretch during movements such as bending and lifting to assist the low back and hip extensor muscles. Moving the flaps from disengaged to engaged mode creates the desired moment arm extension effect. Moving the flaps back into the disengaged mode causes the elastic bands to run along the side of the waist (i.e. along the neutral axis of body in the sagittal plane) and thus to experience negligible displacement during movements (e.g. lifting, walking, stair ascent/descent) and postures (e.g. standing, sitting, crouching). We note that this new dual-mode flap design that utilizes the neutral axis of the body (Zelik, Fine, et al., 2020) differs from the clutch mechanisms used in our previous form-fitting exosuit (Lamers et al., 2017), but they each accomplish the same goal of achieving one mode in which the device stays out of the way (disengaged state) and one that assists the user (engaged state). A physical prototype of this design was fabricated and is shown in fig. 2). In total this extensible exosuit prototype weighs 1.5 kg (fig. 2).

## 5. Case Study Demonstration

A single-subject case study was performed to demonstrate and confirm the mechanical function of the extensible exosuit prototype. The first test (section 5.1) sought to confirm that the extensible exosuit in engaged mode (i.e., extended mechanism) could provide the same torque assistance but with reduced device-to-body forces ($\left\|\vec{F_{T}}\right\|$ on the shoulders and legs) compared to the form-fitting exosuit during a manual lifting task. The second test (section 5.2) sought to to confirm that the user could perform common movements and postures (e.g. walking, carrying, leaning, twisting, sitting) without feeling restricted while wearing the extensible exosuit in disengaged mode. The subject provided written consent prior to testing according to the approved Vanderbilt University Institutional Review Board protocol.

### 5.1. Exosuit Assistance Demonstration

A single subject (female, 64 kg, 1.74 m, 26 yr.) performed a lifting and lowering task while wearing the extensible exosuit vs. the form-fitting exosuit. User and exosuit kinematics and elastic band tension data were collected. The subject performed 8 lifting and 8 lowering movements with a 13 kg box, paced at 15 lifting/lowering movements per minute. The subject performed the task with the extensible exosuit and with the form-fitting exosuit. The elastic band stiffness was adjusted between both exosuit conditions (i.e. different elastic bands were installed on the extensible vs. form fitting exosuit) to ensure that the same peak exosuit torque assistance (*τ*_*exo*_) was provided for both conditions.

Motion capture markers were placed on the following segments to measure their kinematics: the subject’s trunk, the subject’s pelvis, the trunk interface, the extension mechanism, the elastic bands, and the leg interfaces. One of the elastic bands was instrumented with a load cell to measure the trunk force. The trunk force in the non-instrumented elastic band was matched to the instrumented elastic band by matching the slack length of the two elastic bands and confirming with the subject that the tension of the two elastic bands felt equivalent during the movement. Motion capture (Vicon) and load cell (Futek) data were collected synchronously within the same data acquisition system at 200 Hz and 1000 Hz, respectively. Motion and load cell data were low-pass filtered at 6 Hz and 10 Hz, respectively, with a 4^*th*^ order, dual-pass Butterworth filter (Zelik et al., 2014). *τ*_*exo*_ was calculated using motion capture and load cell data collected during the lifting and lowering trials. Motion capture markers placed on the elastic bands and extension mechanism provided orientation data, and the load cell provided the magnitude of force along the elastic band, which enabled us to calculate force vectors $\left(\vec{F_{T}}\right)$ and $\left(\vec{F_{M}}\right)$. Motion capture markers on the pelvis’ anatomical landmarks were used to estimate the location of the L5-S1 joint (Peng et al., 2015). Time series *τ*_*exo*_ was calculated for all trials and cycles using eqs. (1) to (3). Kinematic and kinetic analysis were performed using the Visual3D software package (C-Motion). Time series kinematic and kinetic data were divided into individual cycles using the weight’s vertical position measured via motion capture as the parsing signal, time-normalized to 1000 data points and then averaged across cycles. Peak *τ*_*exo*_, $\vec{F_{T}}$ and $\vec{F_{M}}$ were calculated for individual cycles, and then averaged. The two key outcome metrics were: *τ*_*exo*_ (to confirm assistance magnitudes were similar for each exosuit condition), and $\vec{F_{T}}$ (to confirm that the extensible exosuit reduced device-to-body forces vs. the form-fitting exosuit). We also used the experimental motion capture data to measure the actual design parameters (*x*_1_, *x*_2_ and *y*_2_) that resulted when the prototype was worn by this specific case study participant.

### 5.2. Exosuit Non-Interference Demonstration

Next the subject performed a series of common movement tasks while wearing the extensible exosuit in disengaged mode. The subject performed the following tasks: level treadmill walking, walking while carrying a 13 kg box, stair ascent/descent, sitting, sit-to-stand, twisting at the torso in the coronal plate, leaning left and right in the frontal plane, leaning forward and backward in the sagittal plane. Immediately after completing each movement the subject filled out a questionnaire (see table A.1 in the Appendix) in which they rated how much they felt that the extensible exosuit interfered with the task on a five point Likert scale.

### 5.3. Case Study Results

The extensible exosuit parameters during the lifting and lowering trials (measured using the motion capture data), were 0.15 m for the trunk interface anchoring point (*x*_1_), −0.025 m for the extension mechanism location on the back (*x*_2_), and 0.19 m for the extension mechanism offset (*y*_2_). These parameters differed slightly from our target design criteria (see section 4.1 above), which was not unexpected since these parameters depend on each person’s body dimensions and precisely how the prototype fits onto their body. Nonetheless the parameters were deemed adequate to achieve our proof-of-concept demonstration goals. When disengaged, the extension mechanism protruded < 2 cm away from the body fig. 2.

**Figure 7.**
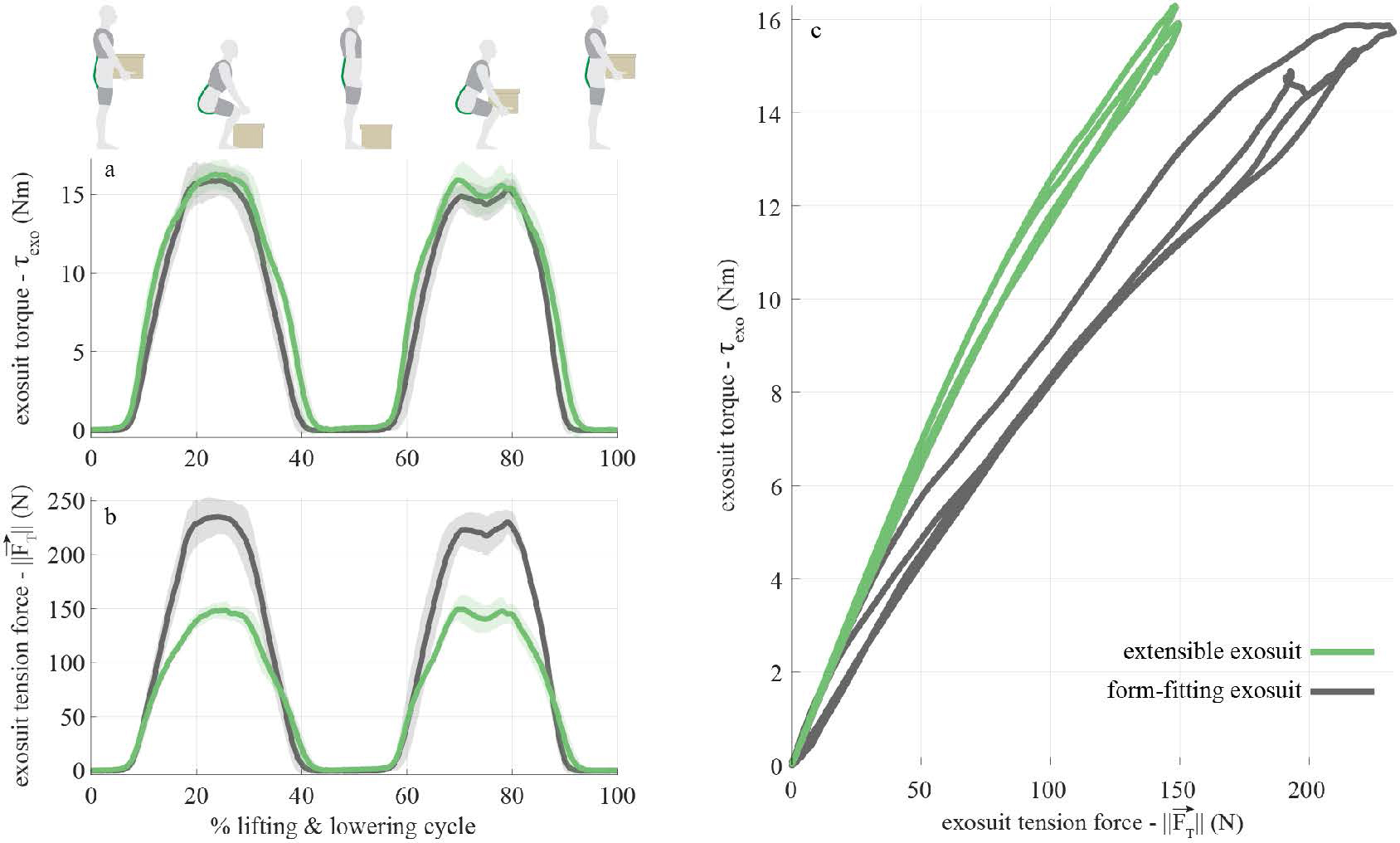
Mechanics of extensible vs. form-fitting exosuit from case study. The extensible exosuit (green curves) provided similar assistance torque (**a**) as the form-fitting exosuit (gray curves), but with lower device-to-body force on the shoulders and legs (**b**, reduced peak force magnitude by 36%). The slopes of the curves in **c** show the relationship between the trunk force magnitude ($\left\|\vec{F_{T}}\right\|$, x-axis) and the assistive torque (*τ*_*exo*_, y-axis). This slope is analytically equivalent to the exosuit moment arm *r*_*T*_. The moment arm *r*_*T*_ for the extensible exosuit (based on a linear least squares fit of each curve) is $0.109\;\frac{Nm}{N}$, which is 63% greater than the slope for the form-fitting exosuit ($0.067\;\frac{Nm}{N}$). Curves in (**a**) and (**b**) depict the mean (solid lines) ± standard deviation (shaded area around mean) across the lifting cycles.

The peak exosuit torques while wearing the extensible and form-fitting exosuits were similar, 17.2 ± 0.5 Nm and 16.7 ± 0.6 Nm, respectively (fig. 7, a). The peak trunk force magnitude for the extensible and form-fitting exosuit were 159 ± 6 N and 249 ± 7 N, respectively (a 36% reduction when wearing the extensible exosuit prototype, fig. 7, b). This reduction was similar to the model predictions when we plugged the measured design parameters (*x*_1_, *x*_2_ and *y*_2_) back into the model. Also of note, this subject reported that they felt the extensible exosuit was more comfortable than the form-fitting exosuit, consistent with the observed reduction in force. For reference, the peak extension mechanism force on the extensible moment arm was 157 ± 7 N. While in disengaged mode, the subject reported that she was able to complete all movement tasks without interference from the extensible exosuit (survey responses are provided in table A.1).

## 6. Discussion

### 6.1. Summary

In this work we developed a human-exosuit biomechanical model which was used to understand how various design parameters affected exosuit assistance torque and device-to-body forces. We used these model findings to inform the design and fabrication of an extensible exosuit prototype. We then demonstrated in a human subject case study that the extensible exosuit could provide the same low back assistance torque as a form-fitting exosuit, but with reduced device-to-body forces on the shoulders and legs (reduced by 36% in the case study, but the model provides insight on how to adjust design parameters to increase or decrease this magnitude as desired). User feedback confirmed that the extensible exosuit successfully provided assistance during lifting, reduced device-to-body forces on the shoulders and legs, improved perceived comfort, and allowed for full freedom of movement and posture (including sitting) when disengaged.

### 6.2. Applications of an Extensible Exosuit

The extensible exosuit offers a way to increase the moment arm of form-fitting exosuits (while in engaged mode), without sacrificing key benefits related to being lightweight, low-profile, and unobstructive (in disengaged mode). The extensible exosuit can reduce device-to-body forces on the shoulders and legs, as shown analytically in the model and confirmed empirically in the case study, which can be used to improve comfort for some users or situations. Alternatively, the extensible exosuit can be used to increase the magnitude of assistance without increasing these device-to-body forces (relative to the form-fitting exosuit), which may be valuable for heavy-lifting jobs. Furthermore, although this exosuit was designed to assist the low back, this extension mechanism concept could be used to assist other joints or segments as well (e.g., ankle, knee, neck or shoulder). An extension mechanism could be used in unpowered (e.g., spring) or powered (e.g., motorized) exosuits to selectively increase the moment arm, or it could be controlled in powered exosuits to actively assist movement (e.g., to inject energy by using a motor to extend the mechanism as the user is lifting).

The dual-mode design detailed here may be well-suited for a variety of occupations and work environments. One worth highlighting is last-mile delivery, which typically involves extended periods of sitting (while driving) and intermittent lifting and carrying. In these types of jobs the ability to shift or rotate rigid/semi-rigid components away from the posterior of the back while in disengaged mode may be beneficial (or critical) to ensure comfort while sitting in delivery vehicles. This style of mode-switching is unique amongst existing back-assist exoskeletons and exosuits, which typically have rigid components along the back or waist that interfere with and may cause discomfort during prolonged sitting. We highlight this application because we are not aware of any commercial or research exoskeletons or exosuits that are particularly well-suited for last-mile delivery, which is a fast-growing market segment. Also of note, this dual-mode flap design can be used with or without an extensible moment arm (i.e. it could also be implemented within a form-fitting exosuit, (Zelik, Fine, et al., 2020)).

### 6.3. Alternative and Future Designs

The goal of this prototype was to demonstrate proof-of-concept of an extensible exosuit. However, there are numerous alternative designs and implementations of an extensible moment arm mechanism (i.e. alternative to the flap design used in this work), such as a four-bar mechanism, an inflatable pneumatic pouch, or a simple hinged lever. Additionally there may be alternative design objectives such as simultaneously increasing the moment arm about multiple joints (e.g., about both the low back and the hip joints), or creating a non-linear assistance torque profile (appendix A.4). These objectives could be achieved by relocating and/or reorienting the extension mechanism, by using multiple extension mechanisms, by changing the shape or trajectory of the extension mechanism, or by adjusting where the elastic bands are affixed along the length of the extension mechanism. Therefore, in addition to increasing the exosuit’s moment arm, an extension mechanism could also be designed to provide a custom torque profile for a given application.

We opted to use the flap extension mechanism design for this proof-of-concept prototype because the design and construction was simple, low-profile (flaps were <6 mm thick), and because the flaps served the dual purpose of mode-switching and extending/collapsing the exosuit extension mechanism. In the future, if we were to build a prototype for the purposes of field testing, then we would upgrade the mode-switching behavior to improve the user experience. The current prototype was sufficient for proof-of-concept but it requires two hands to manually move both flaps from engaged to disengaged position, and vice versa, which hurts usability and user experience. In future iterations this could be simplified by coupling the two flaps such that the user only needs to perform a single movement (e.g. with one hand) to more quickly and easily move the flaps between the engaged and disengaged modes. We have previously built and demonstrated a variety of these mode-switching controls to easily engage and disengage assistance (Lamers et al., 2017); some have used small motors (muscle activity control, voice control, phone app) and others have been purely passive (manual button, switch, knob). The choice of switch is driven by the intended use case of the exosuit.

We achieved our intended goal with this extensible exosuit prototype: we reduced the device-to-body forces on the trunk and legs, while providing the same exosuit assistance torque about the low back (fig. 7). However, the extension mechanism in this prototype did not alter the moment arm with respect to the hip joint. Because the exosuit moment arm about the hip remained the same and the force in the elastic bands was reduced, the assistance torque (and work) about the hips was also reduced with the extensible exosuit, relative to the form-fitting exosuit. Consistent with this biomechanical effect at the hips, the subject reported that they felt like they were getting more assistance during the lift when wearing the form-fitting exosuit. This makes sense: during a squat lifting movement, the lumbar spine undergoes relatively small angular displacement, so the exosuit is primarily providing what we might term a support torque (i.e., reducing force demands on the back extensor muscles which are contracting near-isometrically). In contrast, the hip joints experience large angular displacements during the lifting movement. As the elastic bands stretch and recoil, elastic potential energy is stored and returned to the users as assistive work about the hip joints (i.e., offsetting mechanical work that would otherwise need to be done by the hip extensor muscles). If we were to match tension in the elastic bands instead of matching torque between the extensible and form-fitting exosuits, then we would expect to see the same hip assistance torque (and work) between both exosuits during lifting, but greater L5-S1 support torque in the extensible exosuit. Or if our design goal had been to increase the moment arms about both the L5-S1 and hip joints, then we could have used the same modeling approach we outlined in section 3 to identify the proper exosuit and extension mechanism design parameters to achieve these goals (appendix A.4). This highlights the benefit of using biomechanical modeling to identify design parameters that achieve a specified assistance goal, and also provides a reminder that the prototype demonstrated is simply an example, and that this concept of using an extensible mechanism to increase the moment arm can be adapted to assist one or more body joints or segments.

### 6.4. Additional Model Insights

One insight from the model is that the theoretical upper limit of the moment arm relative to the L5-S1 joint is equal to the distance between the trunk interface anchoring point (*x*_1_) and the L5-S1 joint (i.e. the moment arm *r*_*T*_ ≤ ∥*p*_1_ − *p*_0_∥). This makes intuitive sense: if you imagine an infinitely long extension mechanism (*y*_2_ → ∞), then the force vector on the trunk $\vec{F_{T}}$ would be perpendicular to the trunk segment itself. This configuration is analytically equivalent to a rigid exoskeleton comprised of a rotational spring and a rigid strut from L5-S1 to the trunk interface anchoring point. This is also a useful reminder that despite the common distinction made between rigid exoskeletons and soft exosuits they both operate on the same physical principles. They are springs (or actuators) acting in parallel with the body, and from a physics perspective they represent different sets of parameters along a continuum of possibilities. In a sense, our extensible exosuit concept is a design somewhere in the middle of this continuum, in which we blend some of the benefits of exosuits (e.g. using flexible textiles to minimize weight, movement interference, pressure points, and associated discomfort) with some of the benefits of rigid exoskeletons (e.g. they typically have larger moment arms by nature of applying more perpendicular device-to-body forces farther away from the biological joint center of rotation).

A second interesting model insight is that: when the extension mechanism is placed on the low back, the force $\left(\vec{F_{M}}\right)$ it exerts on the body causes the moment arm to plateau at around 0.22 m, which is considerably lower than the theoretical upper limit of 0.41 m. This holds true even as the extension mechanism offset is increased (e.g. up to 0.58 m, fig. A.3). This happens because $\vec{F_{M}}$ changes orientation as *y*_2_ increases, such that it creates a flexion torque component (clockwise) about the L5-S1 which directly opposes the extension torque component (counter-clockwise) of $\vec{F_{T}}$. This may be a limitation for this particular proof-of-concept design implementation (i.e. mechanism sitting on the low back); however, there are alternative ways to configure the extensible mechanism (or mechanisms) that would create an even larger moment arm (see appendix A.4 for examples of alternatives). However, in practice, this limitation may be moot, because if the extension mechanism becomes too large, then it will be considered by the users as too bulky and impractical to adopt.

A final insight is related to the physical design of the exosuit, and specifically the placement of the elastic elements (materials) within the bands. While the model depicts an elastic material running continuously between the trunk and leg interfaces (fig. 1), fabricating the physical device often requires this band to be comprised of a combination of elastic/stretch and inelastic/non-stretch materials in series (fig. 2). This introduces practical design choices, such as deciding whether the elastic element (within the band) should be located near the middle of the back vs. behind the buttocks vs. behind the legs, etc. For this extensible exosuit prototype (fig. 2) and for our previous exosuit designs (Lamers et al., 2018) we chose to place the elastic elements over the buttocks and the non-stretch webbing over the user’s back. From our own experience in testing and designing these exosuits, we have found that this placement of the elastic elements makes the exosuit subjectively feel more comfortable and assistive to us. To provide insight on this topic we extended our biomechanical model to consider the effects of friction between the exosuit and the user. The model (detailed in appendix A.5) suggests that the benefit of placing the elastic element over the buttocks is that it minimizes relative motion between the band and the buttocks. This in turn minimizes dissipative energy losses due to friction. In contrast, placing an non-stretch material (e.g. non-stretch webbing) over the buttocks results in frictional losses each time a person bends or lifts. Interestingly, most people are already familiar with this physical phenomenon from their own personal life experience: When you bend forward or squat down while wearing non-stretch pants (e.g. denim jeans), your pants tend to slide down in the back (potentially exposing your intergluteal cleft, i.e. your butt crack). Whereas this sliding effect does not happen (or is greatly reduced) when wearing elastic or stretch pants (e.g. spandex leggings) because the elastic fabric deforms with your buttocks as you bend. Consistent with this shared human experience, the key takeaway from our model is that placing the elastic element over the buttocks likely reduces friction force and dissipative work, which may explain why this configuration subjectively feels more comfortable and assistive than alternative configurations (e.g. placing the non-stretch element over the buttocks and the elastic element in the middle of the back). Additional technical details and visual illustrations related to elastic band placement, buttocks friction and dissipative work are provided in appendix A.5.

### 6.5. Scope of Work and Limitations

First, regarding the scope of work, we chose not to assess back muscle activity in the case study. This is because over the last 15 years there have already been over a dozen independent studies consistently showing that these types of exosuits reduce back muscle loading and fatigue during lifting and bending tasks, and also that the magnitude of back offloading scales with the magnitude of exosuit torque assistance (see appendix A.6 for a table summarizing the evidence). To be specific: when in engaged mode, the extensible exosuit presented here is functionally similar to previous exosuits in the way that they provide assistance torque about the low-back. As such, a case study would not meaningfully advance our understanding beyond the current state of knowledge. The innovations here were the extensible/collapsible nature of the moment arm, the novel mode-switching behavior, and the modeling work that better informs the selection of design parameters.

Second, we employed a simple model of the human and exosuit system, which neglects some 3D geometrical details, curvature of the spine, and soft-body mechanics. Despite these assumptions, our model was adequate for its intended purpose: to provide general insight on design parameters which we could use to inform fabrication of a prototype.

Third, we only tested the extensible exosuit on a single-subject. However, this was sufficient for our purposes: to demonstrate proof-of-concept. Future work includes development of a field testable prototype, and multi-user field test evaluation, in particular to better understand user perceptions and preferences across a larger number of people. For instance, while there may be some individuals who prefer the lower shoulder and leg forces afforded by the extensible exosuit, we have anecdotal evidence that suggests others may find the form-fitting exosuit sufficiently comfortable such that they prefer the higher shoulder forces over an additional force on their lower back or waist. Of note, we previously used this same research-development-translation progression (i.e., modeling and feasibility test, followed by field prototype and field testing, followed by technology translation) to translate the form-fitting exosuit into a commercial product (HeroWear Apex). We hope to follow a similar progression with this extensible exosuit, and this manuscript represents the first stage of that progression.

Finally we note that the biomechanical estimates of L5-S1 torque may be susceptible to errors in absolute magnitude, because the L5-S1 joint location was estimated using external reference markers and regression equations derived from cadaveric pelvises (Peng et al., 2015). However, in this work we only look at differences between the extensible exosuit vs. the form-fitting exosuit (i.e., relative differences), which use the same estimated L5-S1 location, and therefore absolute errors in the magnitude of the torque assistance do not affect the relative comparisons or any of our conclusions.

## 7. Conclusion

The dual-mode extensible exosuit introduced here provides a practical and effective way to enhance the moment arm of exosuits, while also retaining key benefits of not interfering with movement and being low-profile while disengaged. A proof-of-concept prototype was demonstrated, and the modeling work provides the foundation for broad applications and various implementations of extensible exosuits to enhance human health and safety, for the back and other body segments. We envision promising opportunities to apply this extensible exosuit concept to assist heavy-lifting, to further enhance user comfort, and to address the unique needs of last-mile delivery workers.

## Figures and Tables

**Table 1.** **Top**: Anthropometric measurements used to scale the model to a 50*^th^* percentile male (Gordon et al., 2016; Jackson et al., 1998). **Bottom**: Domain of the parameters with respect to the L5-S1 joint (coordinate system defined in fig. 3) used for the parameter exploration. The trunk interface anchoring point (*x*_1_) was restricted to sit at or above the L5-S1 (*x*_0_) and at or below the shoulder (*d*_50_). The extension mechanism position along the back (*x*_2_) was restricted to sit at or above the apex of the buttocks (*x*_4_-*r_butt_*) and at or below the shoulder (*d*_50_). The extension mechanism offset (*y*_2_) was restricted to sit at or above the skin surface (*d_skin_*) and at or below 0.2 m offset from the skin surface (note: in a secondary analysis we explored *y*_2_ out to 0.5 m offset from the skin surface and this extended parameter sweep is presented in fig. A.3).

Measurement	Value

*r_butt_*	0.1m
*x*_4_	−0.135 m
*x*_5_	0.4 m
*d_skin_*	0.08 m
Parameter	Minimum	Maximum

trunk interface anchoring point (*x*_1_)	L5-S1 (*x*_0_)	shoulder (*x*_5_)
ext. mech. position (*x*_2_)	buttocks (*x*_4_ - *r_butt_*)	shoulder (*x*_5_)
ext. mech. offset (*y*_2_)	skin surface (*d_skin_*)	*d_skin_* + 0.2 m

**Figure A.1.**
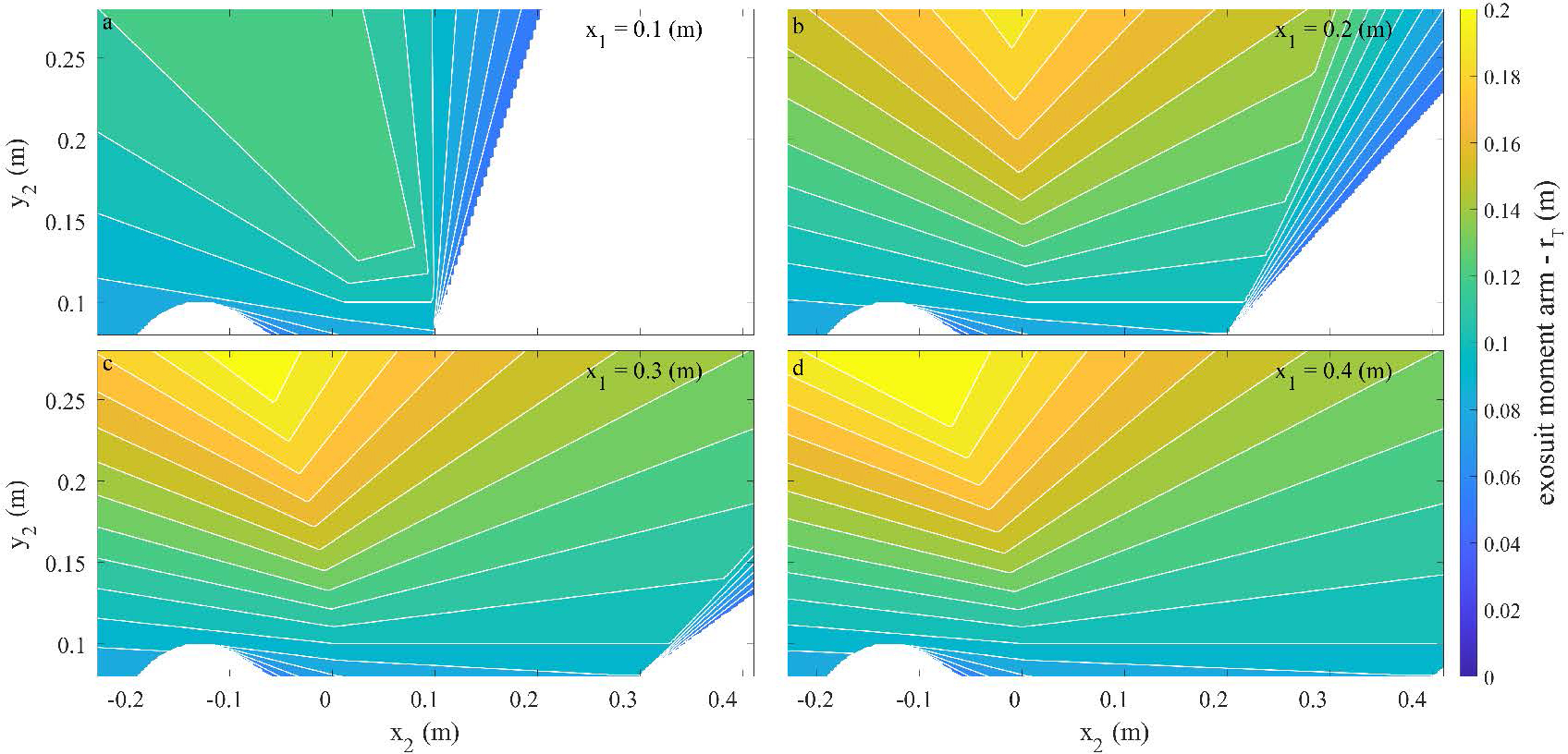
Extensible exosuit moment arm (*r*_*T*_) calculated from eq. (4) across the *x*_2_ and *y*_2_ parameter domain specified in table 1. Each subplot includes a contour plot for a different constant *x*_1_ value (**a**: *x*_1_ = 0.1 m, **b**: *x*_1_ = 0.2 m, **c**: *x*_1_ = 0.3 m, **d**: *x*_1_ = 0.4 m). All points along a contour line denote parameter combinations with a constant *r*_*T*_ in meters. As a reminder, in this model higher values of *r*_*T*_ signify lower device-to-body forces on the shoulders and legs. The x- and y-locations of the routing point *p*_2_ are the axes of the plot (*x*_2_ along the x-axis and *y*_2_ along the y-axis). White regions in the contour plots indicate invalid parameter combinations.

**Figure A.2.**
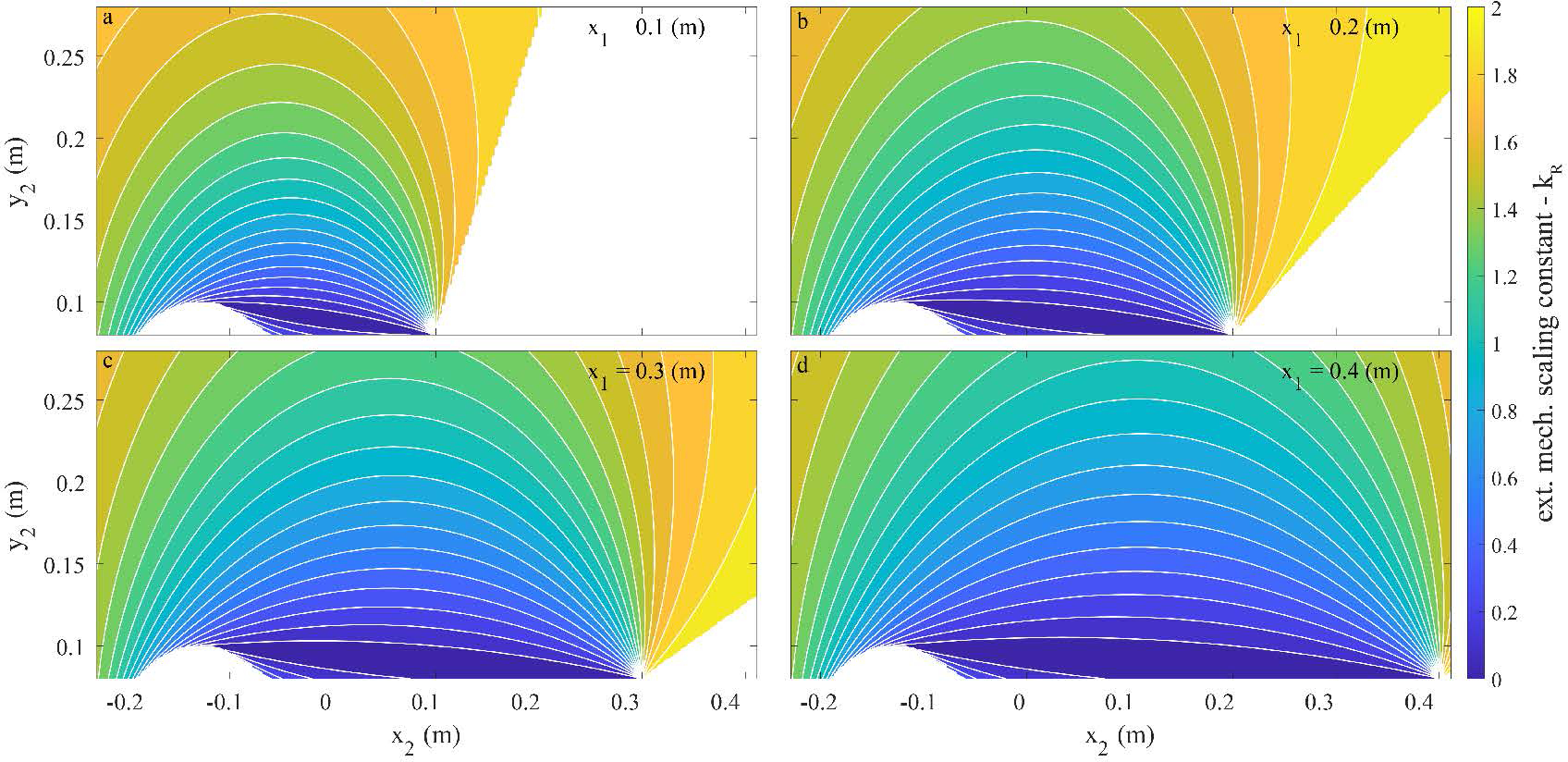
Extensible exosuit *k*_*R*_ calculated from eq. (5) across the *x*_2_ and *y*_2_ parameter domain specified in table 1. Each subplot includes a contour plot for a different constant *x*_1_ value (**a**: *x*_1_ = 0.1 m, **b**: *x*_1_ = 0.2 m, **c**: *x*_1_ = 0.3 m, **d**: *x*_1_ = 0.4 m). All points along a contour line denote parameter combinations with a constant *k*_*R*_. As a reminder, in this model lower values of *k*_*R*_ signify lower device-to-body forces from the extension mechanism onto the back or waist. The x- and y-locations of the routing point *p*_2_ are the axes of the plot (*x*_2_ along the x-axis and *y*_2_ along the y-axis). White regions in the contour plots indicate invalid parameter combinations.

**Figure A.4.**
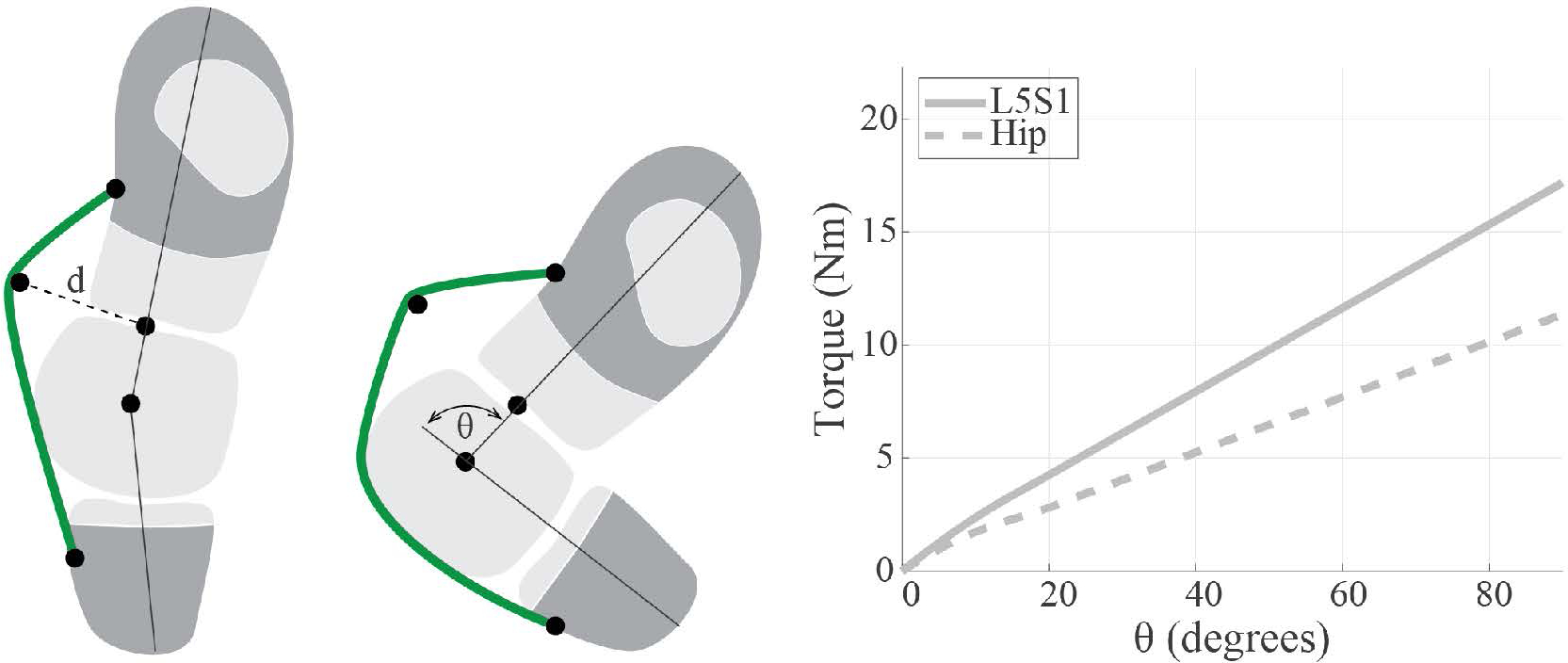
The proof-of-concept extensible exosuit with an extension mechanism on the back/waist (i.e. the design detailed in the main text). This configuration has an increased moment arm about the L5-S1 joint and a linear torque vs. angle profile, with a greater torque about the L5-S1 than the hip. The L5-S1 and hip torque for the extensible exosuit prototype explored in this work are shown here (note we use the same model as discussed in section 3, but expanded the model to estimate the change in torque across a lifting movement). We note that the torque curves for both the L5-S1 and hip are largely linear, and that the torque at the hip is lower than the L5-S1 (because the moment arm is increased at the L5-S1 but not the hip). We include this model as a comparison for the alternative design approaches shown in figs. A.5 and A.6, which use one or more extension mechanism on different locations along the backside.

**Figure A.5.**
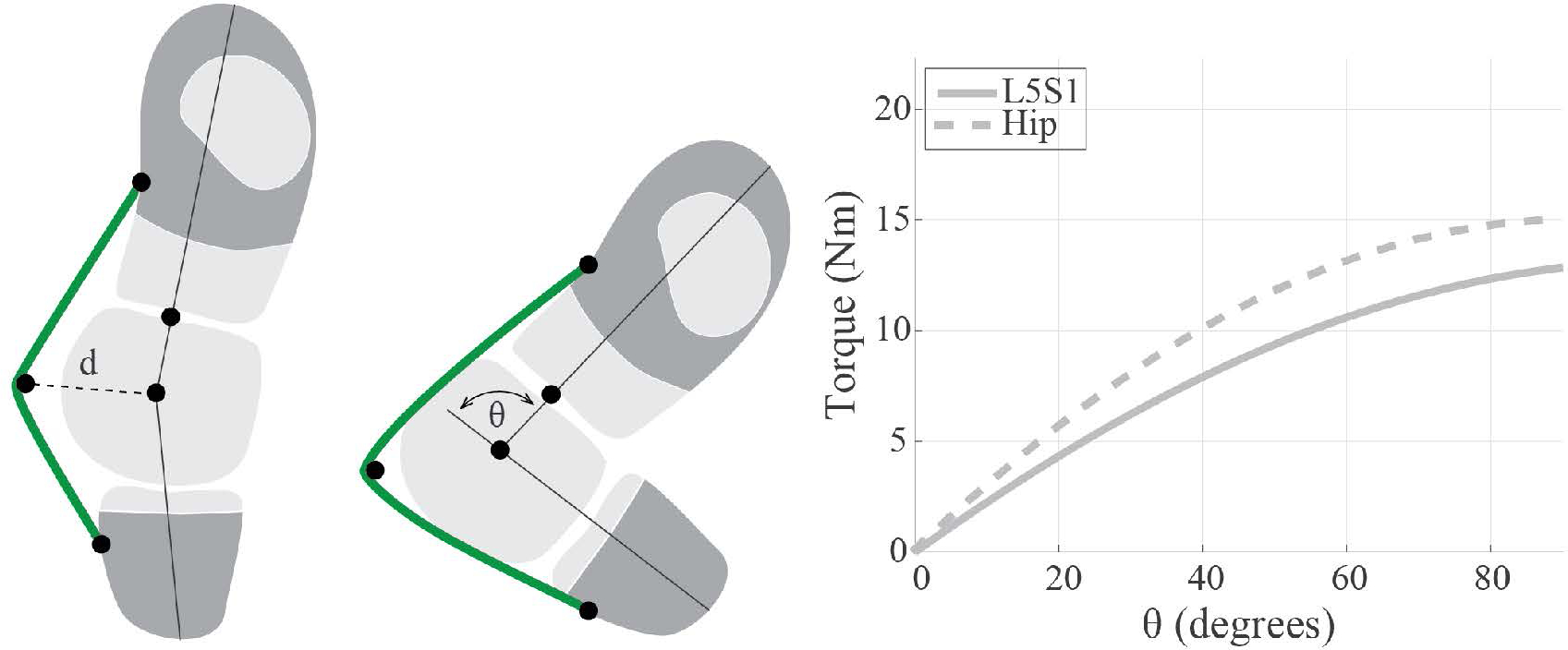
One alternative extensible exosuit design with an extension mechanism on the buttocks creates an increased moment arm and nonlinear torque profiles at both the hip and L5-S1. The geometry of this exosuit changes as the user flexes forward (*θ*), which changes the moment arm of the exosuit with respect to the L5-S1 and hip joints. This also causes a nonlinear displacement of the elastic bands, with a greater torque about the hip than the L5-S1 joint. The result is a nonlinear (softening spring) assistive torque profile for both the hip and the L5-S1 joints.

**Figure A.6.**
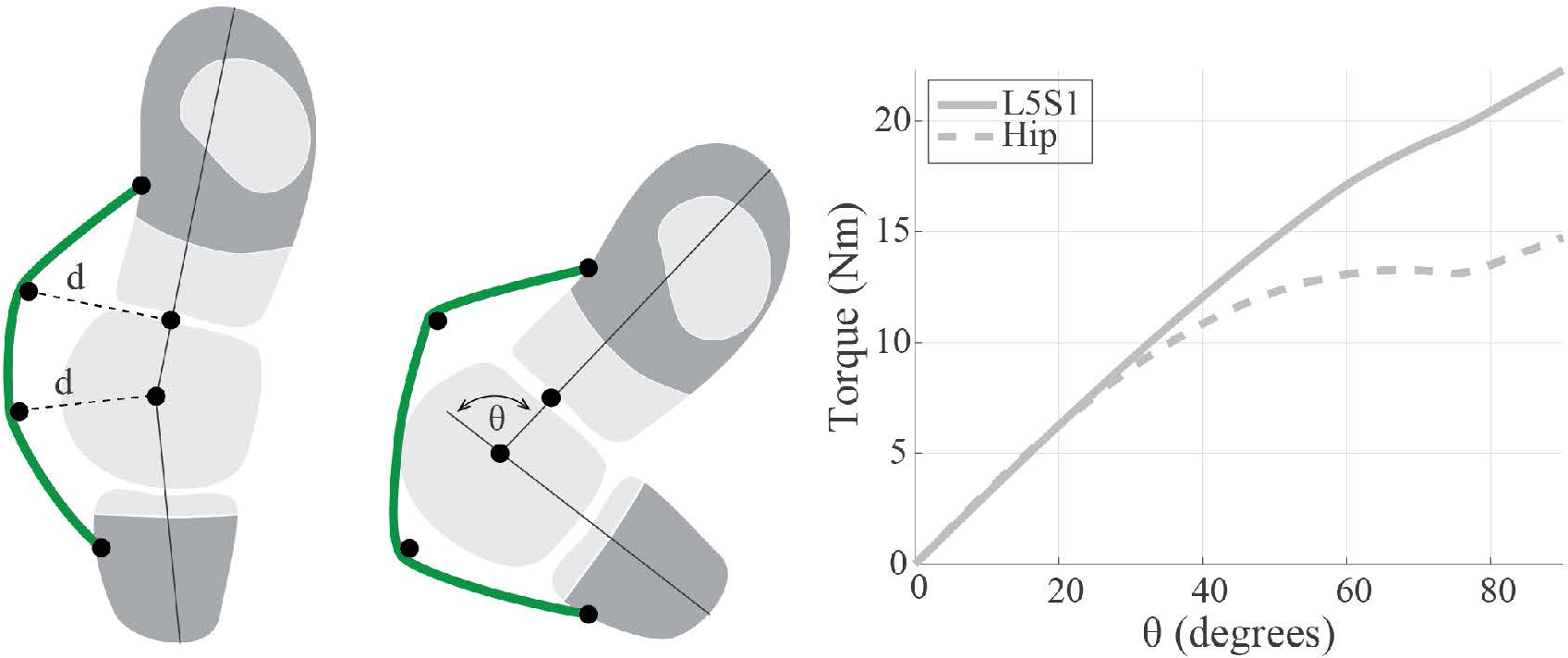
A second alternative extensible exosuit design uses two extension mechanisms. This design includes two offsets, one near the low back and another near the bottom of the buttocks. Compared to the extensible exosuit design tested in the main text of this work, this alternative design increases moment arms relative to both the L5-S1 and hip joints. Additionally, due to the altered geometry, the rate of elastic element displacement is accelerated (relative to fig. A.4) and the hip torque becomes nonlinear. The net effect is that the exosuit torque magnitudes about the hip and L5-S1 are increased relative to fig. A.4.

**Figure A.7.**
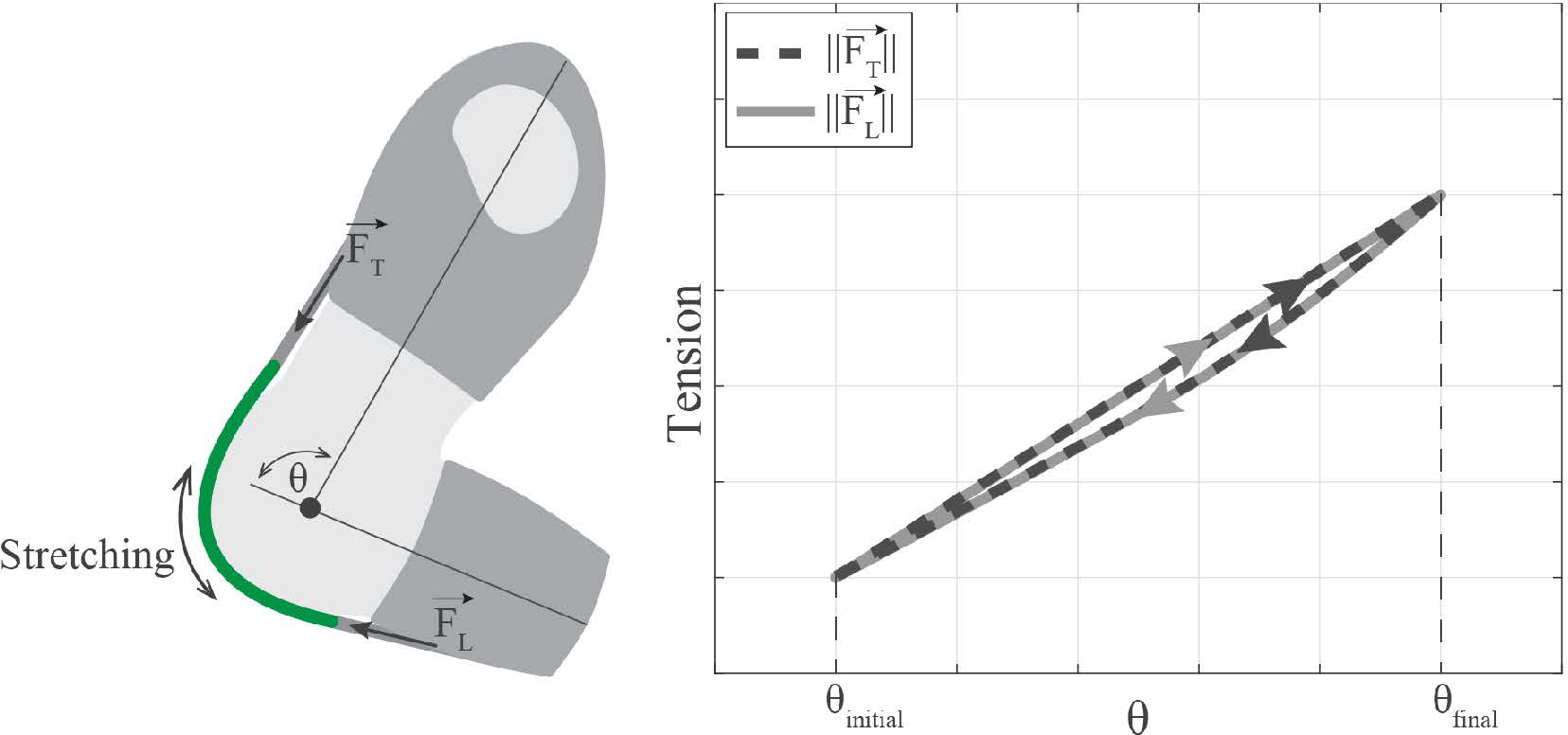
Exosuit with elastic element over the buttocks (green) and non-stretch elements (gray) spanning the back and leg. The elastic element is expected to deform with the buttocks’ change in arc length during squatting or bending, such that there is minimal relative movement between the band and buttocks. As a result, the model suggests that the tension magnitude at the leg $\left(\left\|\vec{F_{L}}\right\|\right)$ and the trunk $\left(\left\|\vec{F_{T}}\right\|\right)$ will be approximately equal. The plot on the right is a qualitative representation to provide intuition on the expected dynamics of the exosuit. In this exosuit configuration energy losses due to butt friction are expected to be minimal.

**Figure A.8.**
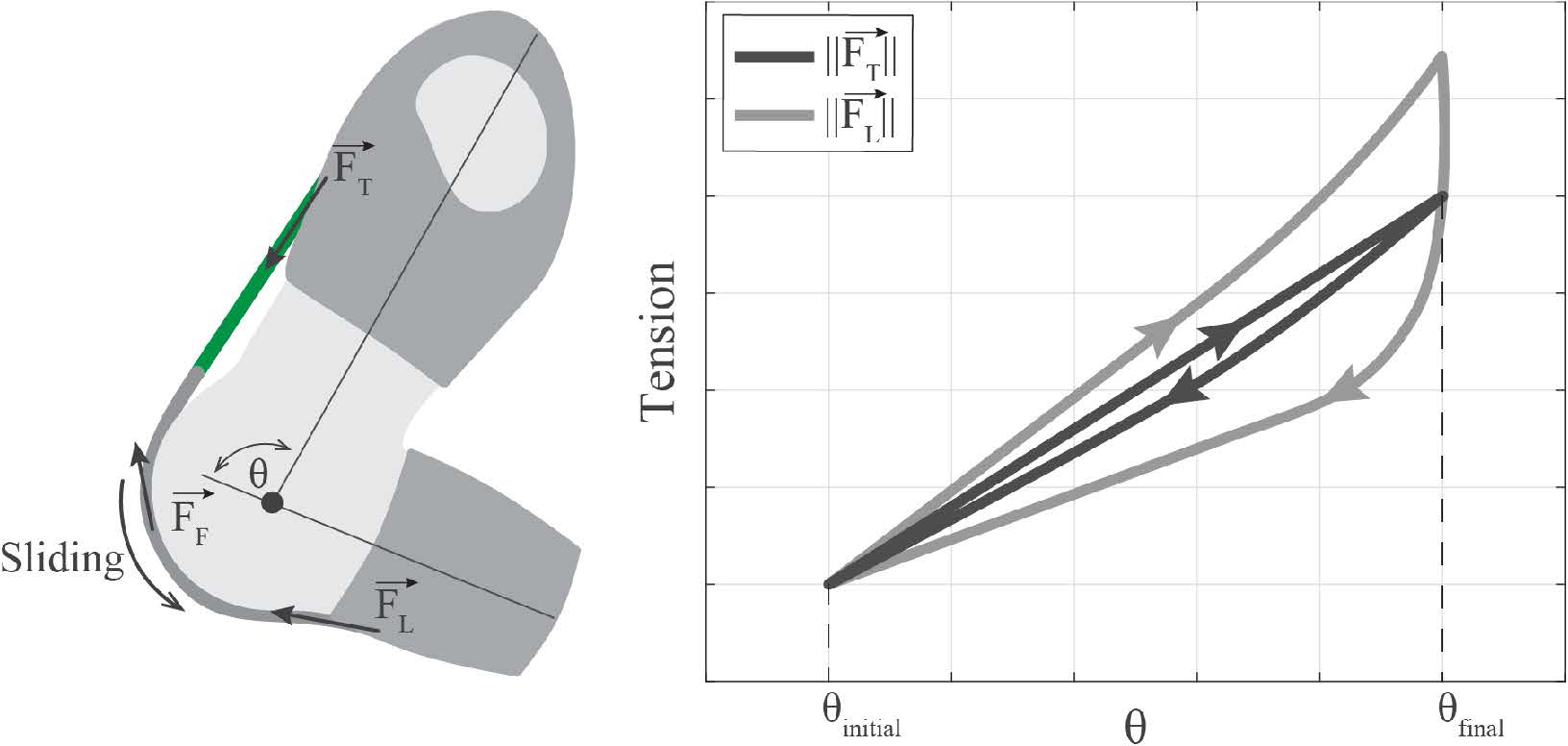
Exosuit with non-stretch element (gray) over the buttocks, and elastic element (green) above it. The model suggests that $\left\|\vec{F_{L}}\right\|$ will be increased during hip flexion (*θ*_*initial*_ → *θ*_*f inal*_), but decreased during hip extension (*θ*_*f inal*_ → *θ*_*initial*_), relative to $\left\|\vec{F_{T}}\right\|$ due to the presence of the frictional force at the butt $\left(\left\|\vec{F_{T}}\right\|\right)$. This difference in tension forces is illustrated by the plot of the light gray curve in the plot on the right. This plot is a qualitative representation to provide intuition on the expected dynamics of the exosuit. In this exosuit configuration energy losses due to butt friction are expected to be non-negligible..

**Figure A.9.**
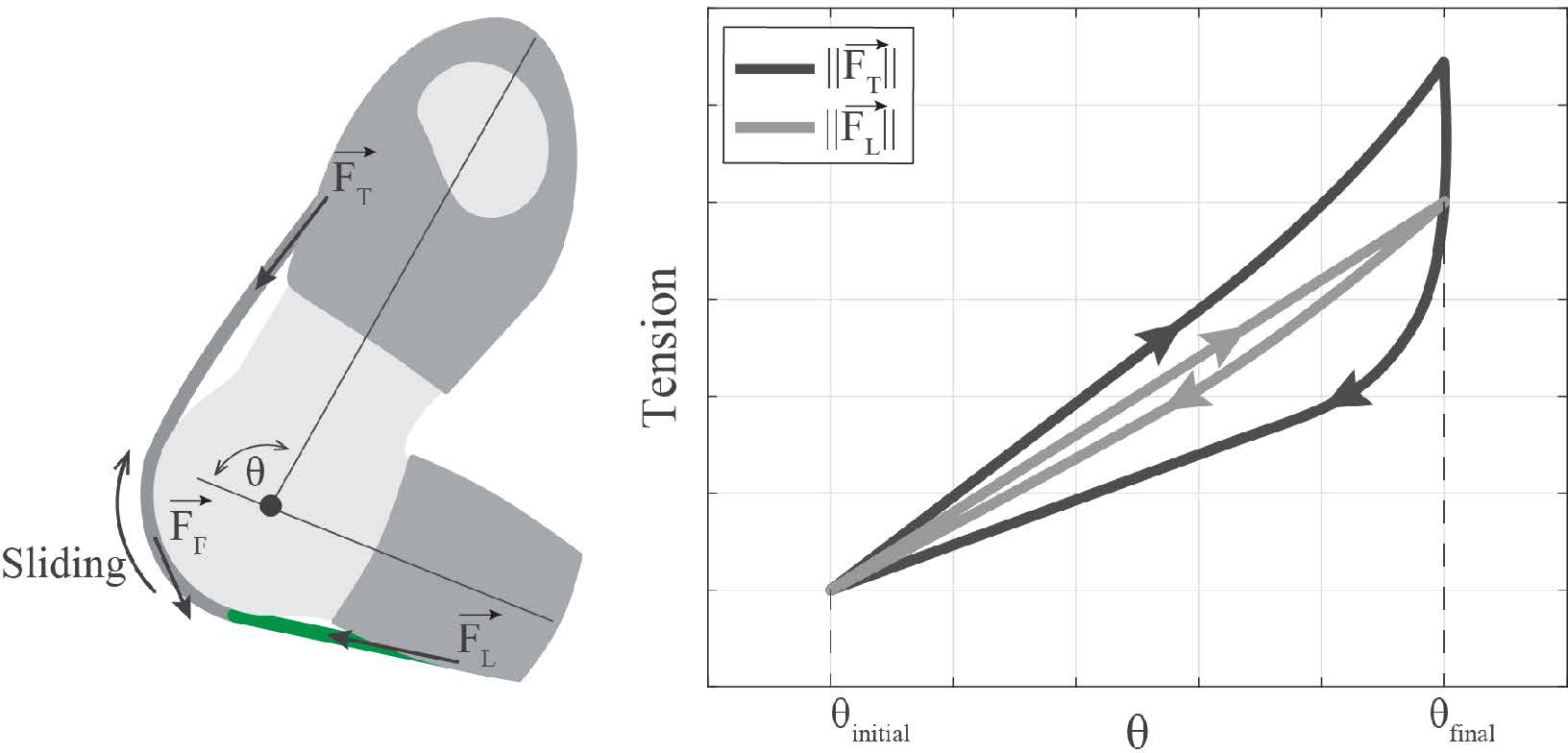
Exosuit with non-stretch element (gray) over the buttocks, and elastic element (green) below it. The model suggests that $\left\|\vec{F_{T}}\right\|$ will be increased during bending (*θ*_*initial*_ → *θ*_*f inal*_), but decreased during lifting (*θ*_*f inal*_ → *θ*_*initial*_), relative to $\left\|\vec{F_{L}}\right\|$ due to the presence of the frictional force at the butt $\left\|\vec{F_{T}}\right\|$. This difference in tension forces is illustrated by the plot of the dark gray curve in the plot on the right. This plot is a qualitative representation to provide intuition on the expected dynamics of the exosuit. In this exosuit configuration energy losses due to butt friction are expected to be non-negligible.

## A. Appendix

### A.1. Subject Feedback From Case Study

**Table A.1.** **Subject survey responses after performing a series of common movement tasks with the extensible exosuit in disengaged mode**. Subject responses are **bold and underlined**.

Sit in chair for ≥ 1 minute: **The exosuit interfered while sitting in the chair**	Strongly Agree	Agree	Neutral	Disagree	**Strongly Disagree**
Sit-to-stand transitions for ≥ 10 cycles: **The exosuit interfered while transitioning between sitting and standing**	Strongly Agree	Agree	Neutral	**Disagree**	Strongly Disagree
Squatting for ≥ 10 cycles: **The exosuit interfered while squatting**	Strongly Agree	Agree	Neutral	Disagree	**Strongly Disagree**
Lifting for ≥ 10 cycles: **The exosuit interfered while lifting**	Strongly Agree	Agree	Neutral	Disagree	**Strongly Disagree**
Leaning forward & backward for ≥ 10 cycles: **The exosuit interfered while leaning forward or backward**	Strongly Agree	Agree	Neutral	Disagree	**Strongly Disagree**
Leaning left & right for ≥ 10 cycles: **The exosuit interfered while leaning to the left or right**	Strongly Agree	Agree	Neutral	Disagree	**Strongly Disagree**
Twisting left & right ≥ 10 cycles: **The exosuit interfered while twisting left or right**	Strongly Agree	Agree	Neutral	Disagree	**Strongly Disagree**
Level walking for ≥ 1 minute: **The exosuit interfered while walking**	Strongly Agree	Agree	Neutral	**Disagree**	Strongly Disagree
Walking & carrying a box for ≥ 1 minute: **The exosuit interfered while walking and carrying a box**	Strongly Agree	Agree	Neutral	Disagree	**Strongly Disagree**
Walking up & down stairs for ≥ 1 minute: **The exosuit interfered while walking up or down stairs**	Strongly Agree	Agree	Neutral	Disagree	**Strongly Disagree**

### A.3. Model Parameter Selection

For our proof-of-concept prototype we initially aimed to design an extensible exosuit that would reduce $\left\|\vec{F_{T}}\right\|$ by about 50% and minimize the exosuit footprint (i.e. minimum extension mechanism offset *y*_2_) for the average male user (e.g. 50^*th*^ percentile). Reducing $\left\|\vec{F_{T}}\right\|$ by 50% is analytically equivalent to doubling *r*_*T*_. We followed this process below to establish target design parameters for the prototype:

- We first scaled the model to a 50^*th*^ percentile male based on anthropometric data (table 1, bottom, (Gordon et al., 2016; Jackson et al., 1998)).
- Next, using the scaled model, we defined our exosuit design parameter ranges (i.e. minimum and maximum values, table 1, top), generated 3D parameter grids (e.g. meshgrid function in MATLAB), and fed these grids into eqs. (4) and (5).
- Next we assumed a baseline *r*_*T*_ (0.08 m) based on our previous work (Lamers et al., 2018). Therefore our desired/target *r*_*T*_ was 0.16 m.
- Next we chose a trunk interface anchoring point (*x*_1_ = 0.2 m) that worked best for our design constraints.
- With *r*_*T*_ and *x*_1_ defined we are constrained to a single contour line (e.g. fig. 4). Along that contour line, we chose the point with the smallest *y*_2_ in order to minimize the footprint of the exosuit. We found these to be *x*_2_ = 0.0 m, *y*_2_ = 0.18 m (fig. 4, black dot).
- The target parameters chosen were *x*_2_ = 0.0 m, *y*_2_ = 0.18 m, *x*_1_ = 0.2 m.

### A.4. Examples of Alternative Extensible Exosuit Designs

In the main text we describe numerous ways to alter the design of the extensible exosuit, for instance, by altering the location and number of extension mechanisms. Ultimately, these choices are driven by the specified goal of the exosuit, based on its intended end-user and use case. The breadth of design possibilities highlights the power of this extension mechanism concept. To make these possibilities less abstract, we provide a few tangible examples. These may provide a better sense of the versatility of this extensible exosuit concept, and elucidate how it can be applied to customize designs that, for instance, create non-linear assistive torque profiles or simultaneously increase the moment arm about multiple joints. For the models shown in figs. A.4 to A.6, the magnitude of the extension mechanism offset (*d*) is the same, as are the model scaling parameters (e.g. *r*_*butt*_, trunk and leg anchoring points). The parameters that changed between the models were the location and/or the number of extension mechanisms.

### A.5. Butt Friction and Dissipative Butt Work

In this section we expound upon model insights related to how the placement of the elastic element within the band affects butt friction and dissipative butt work. For the purposes of this summary section, the term band refers to the entire physical connection between the trunk and leg interfaces. The band is comprised of both elastic (stretch) and non-elastic (non-stretch) elements in series with each other.

We begin with a brief summary of how friction between the buttocks and band is expected to affect exosuit dynamics: The buttocks (and other body segments such as the lower back (Huysamen et al., 2018)) deform in a way that changes it’s surface length (i.e. arc length) during movement (e.g. hip flexion). If the elastic element is positioned over the buttocks (as with the extensible exosuit prototype, fig. 2), then the elastic element will experience the same (or similar) displacement as the buttocks surface, with minimal sliding relative to the buttocks (fig. A.7). However, if instead, the non-stretch element is placed over the buttocks, then this non-stretch material will need to slide relative to the buttocks during movement figs. A.8 and A.9). As a result, placing the non-stretch element over the buttocks introduces friction forces which changes the tension along the length of the band, performs dissipative work and is a potential source of chafing over time. Energy dissipated due to friction would otherwise have gone into assisting a user as they are lifting, or returning from a crouched/stooped posture to a standing posture.

Next we provide a more technical summary of the model predictions, which elucidate how tension in different portions of the band, butt friction and dissipative work are related and expected to change during movement, for instance, during a squat lift.

First let us consider an exosuit design with an non-stretch element over the buttocks and an elastic element positioned over the back. As the user squats down (i.e. hips flexing), the non-stretch element and the buttocks slide relative to each other, causing the band to experience a shearing friction force. This increases the band tension such that $\left\|\vec{F_{L}}\right\|>\left\|\vec{F_{T}}\right\|$ (fig. A.8). Next as the user stands back up (i.e. hips extending), the non-stretch element experiences friction which reduces the band tension such that $\left\|\vec{F_{L}}\right\|<\left\|\vec{F_{T}}\right\|$ (fig. A.8).

Using a simple model based on the Euler-Eytelwein formula (Eytelwein, 1832) for capstan friction, we estimate the expected relationship between the force magnitudes at the trunk and the leg: in the eq. (A.1) below, *r*_*butt*_ is the radius of the buttocks (considered here as the distance from the hip joint center to the buttocks skin surface), *μ*_*butt*_ is the kinetic coefficient of friction between the band and the buttocks (here assumed to be the kinetic coefficient of friction between the exosuit and a clothed user, i.e. fabric-on-fabric), *k*_*elastic*_ is the spring constant of the elastic element, and *θ* is the angle of the leg with respect to the trunk as shown in fig. A.7.

(A.1)
$$\left\|\vec{F_{L}}\right\|=\left\|\vec{F_{T}}\right\|e^{\theta \mu_{butt}sgn(\dot{\theta })}=r_{butt}k_{elastic}\theta e^{\theta \mu_{but\text{t}}sgn(\dot{\theta })}$$

In eq. (A.1) $\left\|\vec{F_{L}}\right\|>\left\|\vec{F_{T}}\right\|$ when the hip is flexing (i.e. $\dot{\theta }>0$), and conversely $\left\|\vec{F_{L}}\right\|<\left\|\vec{F_{T}}\right\|$ when the hip is extending (i.e. $\dot{\theta }<0$) for the case shown in fig. A.8. Functionally this means that when the non-stretch element is positioned over the buttocks (as shown in fig. A.8), a higher exosuit torque is created about the hip when it is flexing (bending down), but a lower exosuit torque is created about the hip when extending (standing up), and the L5-S1 exosuit torque remains unchanged relative to the configuration in fig. A.7 where the elastic element is over the buttocks.

Second, consider an exosuit design where the non-stretch element is still positioned over the buttocks but the elastic element is over the leg (as shown in fig. A.9). For this configuration, eq. (A.1) is adjusted by swapping $\left\|\vec{F_{L}}\right\|$ and $\left\|\vec{F_{T}}\right\|$. In this new configuration (as shown in fig. A.9), a higher exosuit torque is created about the L5-S1 joint when the hips are flexing (bending down), because $\left\|\vec{F_{T}}\right\|>\left\|\vec{F_{L}}\right\|$. But a lower exosuit torque is created about the L5-S1 joint when the hips are extending (standing up), because $\left\|\vec{F_{T}}\right\|<\left\|\vec{F_{L}}\right\|$. Meanwhile the exosuit hip torque remains unchanged relative to the configuration in fig. A.7 where the elastic element is over the buttocks.

Third, consider an exosuit design where the elastic element is positioned over the buttocks, and the non-stretch elements are only located below the trunk interface and above the leg interface (fig. A.7). Since the elastic element deforms and stretches with the buttocks throughout the bending and lifting cycle the frictional effects are negligible in the model, and therefore the exosuit torque generated about the L5-S1 and hip joints during bending and lifting follow similar profiles.

In the configurations in which the non-stretch element is positioned over the buttocks, the butt friction forces are expected to dissipate energy as the user moves (due to the relative motion between the band and buttocks). This dissipated energy, or butt friction work (*W*_*butt f riction*_) can be estimated by the model (eq. (A.2)) over a full flexion/extension movement cycle (*θ*_*initial*_ → *θ*_*f inal*_ → *θ*_*initial*_):

(A.2)
$$W_{butt\;friction}=-r_{butt}^{2}k_{elastic}\left(\int_{\theta_{initial}}^{\theta_{final}}\theta \left(e^{\theta \mu_{butt}}-1\right)d\theta +\int_{\theta_{final}}^{\theta_{initial}}\theta \left(e^{-\theta \mu_{butt}}-1\right)d\theta \right)$$

From eq. (A.2) we can see that for a given cycle (*θ*_*initial*_ → *θ*_*f inal*_ → *θ*_*initial*_) the magnitude of dissipative butt friction work increases exponentially with *θ* (the angle of the leg relative to the trunk). This means the dissipative work due to butt friction is expected to increase significantly as a person bends or crouches more deeply. Another insight from this equation is that dissipative butt friction work is proportional to butt radius squared (i.e. $r_{butt}^{2}$). This suggests an interesting potential trade-off when using an exosuit with an non-stretch element over the buttocks: On one hand, a larger butt radius provides a benefit because it increases the moment arm by which the tension in the exosuit band can provide torque assistance about the hips and back. On the other hand, the biomechanical drawback to a larger butt radius is that it introduces more dissipative energy losses due to friction, per eq. (A.2). The solution suggested by this model is simply to place the elastic element over the buttocks (instead of the non-stretch element), which enables the exosuit to retain it’s moment arm benefit due to butt radius, but without incurring the proportional increase in dissipative butt friction work.

This modeling is presented to provide general insight and physics-based expectations that help inform exosuit design. There are various model limitations, similar to those discussed in the main text. Although the model takeaways seem to make sense and match our intuition and prior experiences, these takeaways should be treated as predictions/expectations, which still require empirical confirmation in the future.

### A.6. Table of Evidence for Back-Assist Exosuits

**Table A.2.** Summary of prior modeling, laboratory, and field-based evidence from the last 15 years showing that these types of exosuits reduce back muscle activity, muscle strain, muscle fatigue, spine compression, and perceived exertion during lifting, bending, leaning, and stooping tasks. Each of the studies summarized below tested a device/prototype that is functionally similar to the extensible or form-fitting exosuit in the engaged mode.

Year	Source	Peer Reviewed	Findings
2005	Abdoli-Eramaki et al. (Clinical Biomechanics)	Yes	Exosuit reduced integrated EMG of lumbar and thoracic erector spinae by 14% to 28% respectively during stoop, squat, and free lifting techniques (5, 15, and 25 kg). N=9 (all male)
2007	Abdoli-Eramaki et al. (J Biomechanics)	Yes	Exosuit provided 23–36 Nm of torque and reduced spine compression and shear forces by an estimated 23–29% and 8–9% respectively during lifting tasks. Mathematical proof (using simplified free body diagrams and two-dimensional moment balance equations) explains how and why this type of passive exosuit reduces loading on back extensor muscles and spine. N=9 (all male)
2008	Graham MS Thesis (Queen’s University)	No	Subjects reported feeling positive assistance from exosuit in automotive assembly task, 8/10 would wear device every day.
2009	Lotz et al. (J Electromyography & Kinesiology)	Yes	Exosuit reduced rate of back muscle and cardiovascular fatigue (EMG RMS, median frequency, perceived exertion ratings, endurance time) during cyclic lifting. N=10 (all male)
2009	Abdoli-Eramaki (US Patent, 7,553,266B2)	No	Exosuit reduced hamstring and low back muscle activity.
2009	Frost et al. (J Electromyography & Kinesiology)	Yes	Increasing stiffness in exosuit elastic bands resulted in greater assistive magnitude, and greater reductions in erector spinae activity (up to 38%). The relationship between elastic band stiffness and EMG reduction appeared to be linear and comparable between different lifting styles (stoop, squat, freestyle). N=13 (all male)
2009	Godwin et al (IJIE)	Yes	Exosuit significantly reduced fatigue for all subjects during 45-min lifting session. N=12 (all female)
2011	Fick MS Thesis (Queen’s University)	No	Overwhelmingly positive feedback on perceived assistance from exosuit during field tests.
2011	Sadler et al. (Ergonomics)	Yes	Exosuit significantly reduced lumbar & thoracic flexion and significantly increase hip and ankle flexion (for both males & females). Results suggests exosuit encouraged safe lifting practices without adversely affecting lifting technique (N=30).
2014	Whitfield et al. (IJIE)	Yes	Exosuit reduced thoracic erector spinae and biceps femoris muscle activity by 8% and 14% respectively during box lifting. Exosuit had no significant effect on metabolic rate. N=15 (all males).
2018	Zelik, Yandell, Howser and Lamers (PCT Patent App, WO2018136722A1)	No	Form-fitting exosuit reduced low back muscle activity during bending and lifting.
2018	Lamers, Yang & Zelik (IEEE TBME)	Yes	Exosuit reduced average erector spinae activity 23–43% during leaning, and 14–16% during bending. Peak EMG reduced by 19–23% during lifting (N=8). Physics model (using a simple moment balance) indicated that offloading the low back muscles with this type of exosuit is also expected to reduce intervertebral disc compression forces. Model indicates the reduction in loading of back muscles and discs is expected to increase with both increasing elastic band force and increasing moment arm. N=8, (7 male, 1 female)
2019	Galiana et al. (PCT Patent App WO2019161232A1)	No	Exosuit reductions in EMG: 20–40% for biceps femoris, 20–30% for gluteus maximus, 15–30% for lumbar erector spinae, 40–60% for thoracic erector spinae during leaning (N=1).
2019	Swissport (IATA Ground Handling Conference Presentation)	No	Exosuit reduced erector spinae by 15%, multifidus by 30%, gluteus maximus by 50%, and biceps femoris by 10% (N=unknown).
2020	Yandell et al. (WeRob Conference Paper)	Yes	Exosuit reduced average and peak back muscle activity in this field test by ∼10% during lifting/lowering tasks. Two thirds of workers exhibited reductions >15%. >90% of workers reported feeling assisted and that the exosuit made lifting easier.
2020	Lamers et al. (Nature Scientific Reports)	Yes	Five of 6 subjects showed reductions in back muscle fatigue rate (26–87% for individual muscles) during sustained leaning. Average reduction in back muscle fatigue was 30–40 across all muscles and study participants. N=6 (4 male, 2 female)
